# Addressing the Underestimated Burden of RSV in Older Adults in Europe: Epidemiology, Surveillance Gaps, and Public Health Implications

**DOI:** 10.3390/vaccines13050510

**Published:** 2025-05-12

**Authors:** Floriana D’Ambrosio, Marta Lomazzi, Michael Moore, Ada Maida, Roberto Ricciardi, Ludovica Munno, Monia Lettieri, Elisabetta De Vito, Walter Ricciardi, Giovanna Elisa Calabrò

**Affiliations:** 1 Section of Hygiene, Department of Life Sciences and Public Health, Università Cattolica del Sacro Cuore, L.Go F. Vito 1, 00168 Rome, Italy; florianadambrosio@libero.it (F.D.); ada.maida01@icatt.it (A.M.); ludovica.munno01@icatt.it (L.M.); monialettieri9@gmail.com (M.L.); walter.ricciardi@unicatt.it (W.R.); 2 World Federation of Public Health Associations, ch des Mines 9, 1202 Geneva, Switzerland; mimomph@gmail.com; 3 Institute of Global Health, University of Geneva, ch des Mines 9, 1202 Geneva, Switzerland; 4 VIHTALI (Value in Health Technology and Academy for Leadership & Innovation), Spin-Off of Università Cattolica del Sacro Cuore, 00168 Rome, Italy; robertoricciardi.mail@gmail.com (R.R.); giovannaelisa.calabro@unicas.it (G.E.C.); 5 Department of Human Sciences, Society and Health, University of Cassino and Southern Lazio, 03043 Casino, Italy; elisabetta.devito@unicas.it

**Keywords:** Respiratory syncytial virus, RSV vaccination, at-risk adults, elderly, surveillance systems, epidemiology, Europe

## Abstract

Background/Objectives: Respiratory Syncytial Virus (RSV) is a leading cause of Lower Respiratory Tract Infections (LRTIs), posing a serious threat to vulnerable populations. Although growing evidence highlights its significant impact on older adults, RSV surveillance and data collection remain largely focused on children, underestimating the burden in older and high-risk adults. This review aims to synthesize current evidence on the epidemiological and clinical impact of RSV in older adults in Europe, assess existing surveillance strategies, and identify gaps to guide targeted public health responses. Methods: A two-phase research strategy was adopted. First, a comprehensive review of studies published between 2015–2025 was conducted via PubMed, focusing on the RSV burden in high-risk and elderly populations in Europe. Second, a structured web screening was performed to assess the status of existing RSV surveillance systems, focusing on eight selected European countries. Results: The review reported RSV prevalence rates ranging from 1% to 64.7% among older adults, with a high prevalence of comorbidities that exacerbate disease severity. Hospitalization rates varied between 12.6–55.9%, while mortality ranged from 2.15% to 13%, reaching up to 36% in intensive care settings. Surveillance systems for adult RSV infections across Europe remain limited and fragmented, with only 37.5% (3/8) of analyzed countries having dedicated surveillance for adults. Conclusions: RSV represents a substantial and underrecognized threat to older adults, with significant clinical and healthcare implications. Strengthening surveillance, standardizing data collection, and ensuring equitable access to newly available preventive measures are urgent priorities to reduce the disease burden, protect vulnerable populations, and support resilient health systems against future health challenges.

## 1. Introduction

Respiratory Syncytial Virus (RSV), first isolated in 1956 [1], is currently recognized as a leading global cause of Lower Respiratory Tract Infections (LRTIs), significantly affecting vulnerable populations such as infants, young children, older adults, and individuals with underlying medical conditions [2,3].

Historically, research and public health efforts have predominantly focused on the impact of RSV infections in pediatric populations. However, in recent years, there has been growing recognition of the substantial burden RSV places on older adults and other at-risk adult groups [4].

The global aging population further exacerbates the challenges associated with RSV. Older adults are particularly susceptible due to age-related immune decline (immunosenescence) [5] and the presence of comorbidities, and this demographic shift is expected to drive an increased burden on healthcare systems and socioeconomic structures [4].

Globally, RSV affects approximately 64 million people each year and is responsible for around 160,000 deaths, making it the second leading cause of mortality after malaria [6,7,8]. Among adults, RSV causes between 60,000 and 160,000 hospitalizations annually in those aged 65 and older, leading to 6000 to 10,000 deaths each year [4,9].

The annual incidence of RSV infection is estimated to range from 3% to 7% in healthy older adults and from 4% to 10% in high-risk groups, based on prospective cohort studies conducted in the United States and Europe [10,11,12]. In the European Union, RSV is responsible for over 158,000 hospitalizations among adults each year, with 92% occurring in individuals aged 65 and older [13].

In 2015, an estimated 1.5 million cases of RSV-related Acute Respiratory Illness (RSV-ARI) occurred among older adults in industrialized countries, with 14.5% requiring hospitalization and around 14,000 in-hospital deaths globally [2].

A more recent meta-analysis estimated that in 2019, about 5.2 million confirmed RSV-ARI cases occurred among adults aged ≥60 years in high-income countries, resulting in 470,000 hospitalizations and 33,000 in-hospital deaths [14]. Conversely, in developed countries, the annual incidence of LRTIs due to RSV is 7.03% among high-risk adults and 4.66% among older adults [15].

Although RSV symptoms in adults are generally milder than in children, severe cases are not uncommon, especially among those with underlying conditions such as immunodeficiency, cardiopulmonary diseases, frailty, as well as those residing in long-term care facilities [16].

The clinical presentation of RSV ranges from mild cold-like symptoms to severe respiratory illness, with complications similar to those caused by influenza and other respiratory viruses. These complications may include pneumonia, intensive care unit (ICU) admission, and the need for mechanical ventilation, with 10–31% of severe cases requiring intensive care and 3–17% necessitating mechanical ventilation [17,18].

Additionally, RSV can also exacerbate pre-existing chronic conditions such as chronic obstructive pulmonary disease (COPD), congestive heart failure (CHF), and asthma, potentially triggering acute events like heart failure, myocardial infarction, and stroke [13,19].

Beyond its epidemiological and clinical burden, RSV also imposes a substantial economic impact. In the United States alone, RSV-related healthcare costs exceed $1 billion per year, with older adults accounting for the majority of hospitalizations and associated expenditures [11,19,20].

Despite its substantial impact, the true burden of RSV in older adults remains poorly understood due to limited routine testing and the absence of standardized surveillance protocols, limiting the comparability of data across countries and the assessment of healthcare burdens.

To address these challenges, the World Health Organization (WHO) launched a 2018 Global Influenza Program pilot to standardize RSV data collection in 14 countries. While promising, this initiative excludes older adults, leaving critical gaps in understanding and hindering policy development [21]. In fact, despite growing evidence highlighting the significant role of RSV in the elderly, most RSV surveillance systems globally, as well as in Europe, continue to focus predominantly on children, leaving older adults underrepresented [22].

The urgency of addressing these gaps has intensified with the availability of RSV vaccines for elderly and at-risk adults, underscoring the critical role of vaccination in mitigating the burden of RSV in older populations [23]. Accurate and comprehensive data are essential for assessing the real-world impact of vaccination programs, informing of public health policies, and optimizing prevention strategies [24]. Reliable surveillance systems play a crucial role in quantifying vaccine effectiveness, monitoring disease trends, and ensuring that high-risk populations receive adequate protection [23]. After decades of developments, three RSV vaccines are now available and approved by the U.S. Food and Drug Administration (FDA) and the European Medicines Agency (EMA): two protein subunit vaccines (GSK’s Arexvy and Pfizer’s Abrysvo) and one mRNA RSV vaccine (Moderna’s mRESVIA) [25]. Specifically, RSVPreF3 (Arexvy, GSK) has been approved by regulatory bodies for the following indications: adults aged 50–59 years at increased risk of RSV disease, and all adults aged ≥60 years; RSVPreF (Abrysvo, Pfizer) has been approved by FDA for adults aged 18–59 years at increased risk of severe disease; all adults aged ≥60 years, and pregnant individuals at 32–36 weeks of gestation to protect infants from birth up to 6 months. Instead, in Europe it has been approved for all adults aged ≥60 years and for pregnant individuals at 24–36 weeks of gestation. Finally, mRNA-1345 (mRESVIA, Moderna) has been approved by FDA and EMA for all adults aged ≥60 years [25].

The availability of these vaccines offers new, tailored opportunities to prevent severe RSV-related disease. Several countries have already incorporated RSV vaccination into their national immunization programs for older adults, including Austria, Belgium, Germany, Sweden, and the United Kingdom (UK) [26,27].

In parallel, the field of prevention continues to expand, supported by a growing pipeline of RSV vaccine candidates based on diverse technological platforms, such as messenger ribonucleic acid (mRNA), live-attenuated (LAV), subunit, and recombinant vector-based vaccines targeting different viral proteins [28]. In the coming years, additional immunizing agents are expected to gain market approval, supported by data from ongoing clinical trials [28]. Notably, early real-world effectiveness data in older adults are already available from the first RSV season following vaccine approval, and co-administration studies with other vaccines have been conducted for some candidates, with further research planned to explore potential immune interactions [28]. For example, a study conducted in the U.S. during the 2023–2024 RSV season demonstrated that vaccination was effective in preventing RSV-associated hospitalizations and emergency department (ED) visits among adults aged ≥60 years [29].

However, despite the availability of effective vaccines, the lack of robust collection data and limited awareness continue to hinder the establishment of effective surveillance systems and policies, ultimately undermining efforts to protect at-risk populations.

This review seeks to highlight the significant impact of RSV-related diseases on older adults, a population often overlooked in surveillance and prevention initiatives. By systematizing the available evidence on the epidemiological burden of RSV among older adults in Europe, the review provides an in-depth analysis of the current RSV surveillance strategies and existing data-collection systems. While the primary focus is on Europe as a whole, a more detailed assessment of surveillance systems was conducted for eight selected countries (Bulgaria, Finland, Germany, Italy, Norway, Portugal, Serbia, Spain). The selection of these countries is driven by the interest in analyzing diverse healthcare systems, as well as countries with varying levels of health investment and different stages of discussion or progress in RSV-related policies. Its final objective is to identify the main challenges related to the proper monitoring of RSV infections in the European adult and older population in order to support policymakers in the development and implementation of targeted prevention strategies, aimed at reducing the overall burden of RSV, with a particular focus on the sustainability of health systems.

## 2. Materials and Methods

This assessment and evidence review represent the first step of a study conducted by the World Federation of Public Health Associations (WFPHA) [30].

The evidence collection process was carried out in two phases: the first phase involved a comprehensive literature review to gather relevant studies and data, while the second phase focused on web-screening to assess the current RSV surveillance systems for adult populations. The methodological details of both phases are outlined below.

### 2.1. Literature Review: Search Strategy and Inclusion/Exclusion Criteria

To assess the epidemiological and clinical burden of RSV among elderly and high-risk adult populations in Europe, a comprehensive review of the published scientific literature was conducted.

Although this review does not meet the criteria for a formal systematic review, it followed the guidelines outlined by the Preferred Reporting Items for Systematic Reviews and Meta-Analyses (PRISMA) guidelines [31].

The PubMed database was searched for English-language original articles and systematic reviews published over the past 10 years, with data collected up to 19 February 2025.

The key search terms included “Respiratory syncytial virus”, “RSV”, “adults”, “elderly”, and “Europe”, ensuring the inclusion of studies that specifically focused on high-risk adult and elderly populations within the 27 European Union member states (Austria, Belgium, Bulgaria, Croatia, Cyprus, Czech Republic, Denmark, Estonia, Finland, France, Germany, Greece, Hungary, Ireland, Italy, Latvia, Lithuania, Luxembourg, Malta, Netherlands, Poland, Portugal, Romania, Slovakia, Slovenia, Spain, and Sweden).

For the purpose of this review, “older adults” were defined as individuals aged 60 years and above, consistent with the target population for which the first three RSV vaccines have been authorized [25].

Exclusion criteria encompassed commentaries, editorials, conference presentations, and references lacking full text, as well as studies that did not provide data specific to Europe, were not written in English, or were conducted on animals or in vitro.

### 2.2. Literature Review: Selection Process and Data Synthesis

The initial screening of articles was performed based on titles and abstracts, followed by a full-text review of the papers deemed eligible. A snowballing approach was also employed, where the bibliographic references and citations of selected articles were examined to identify additional studies that met the inclusion criteria.

To ensure the reliability of the included studies, four reviewers independently assessed the articles identified during the literature search (F.D.A., A.M., M.L., and L.M.). Any disagreements were resolved through discussion with a senior researcher (G.E.C.).

The synthesis of the studies was based on their focus and outcomes. Specifically, for studies related to the epidemiological burden, data on the prevalence and incidence of RSV were extracted, with stratification by age or risk factors where possible. Conversely, for studies on the clinical burden, data regarding RSV-related comorbidities, sequelae, hospitalizations, and mortality were extracted and synthesized.

### 2.3. Web-Screening on RSV Surveillance Systems for Adult Populations in Europe

Following the literature review, a structured web-based screening was conducted to collect information on international and national RSV surveillance systems currently in place to monitor RSV infections in high-risk and elderly populations.

This search was conducted by consulting major international and national sources, including the World Health Organization (WHO) [32], the European Centre for Disease Prevention and Control (ECDC) [33], and various national health authority websites.

The focus was specifically on the European context, with an emphasis on eight countries selected for their participation in the aforementioned WFPHA project.

Keywords such as “RSV surveillance”, “RSV infections”, “adults” and “elderly” were used in the query boxes of the consulted websites. Data were retrieved in English and, where available, in the native languages of the selected countries. The data extracted from these sources included information on the surveillance systems in place (e.g., reporting systems, surveillance methods), and their specificities related to RSV infections in adult and elderly populations.

The collected data were then analyzed to identify best practices, existing gaps, and potential opportunities to enhance RSV surveillance efforts, particularly those targeting the elderly population.

## 3. Results

### 3.1. Assessing the Impact of RSV in High-Risk Adults and the Elderly in Europe: A Comprehensive Literature Review

The initial database search identified a total of 386 records. After screening titles and abstracts, 108 full-text articles were selected for further evaluation. Following a thorough assessment, 40 articles met the final inclusion criteria [11,14,18,22,34,35,36,37,38,39,40,41,42,43,44,45,46,47,48,49,50,51,52,53,54,55,56,57,58,59,60,61,62,63,64,65,66,67,68,69].

**Figure 1:**
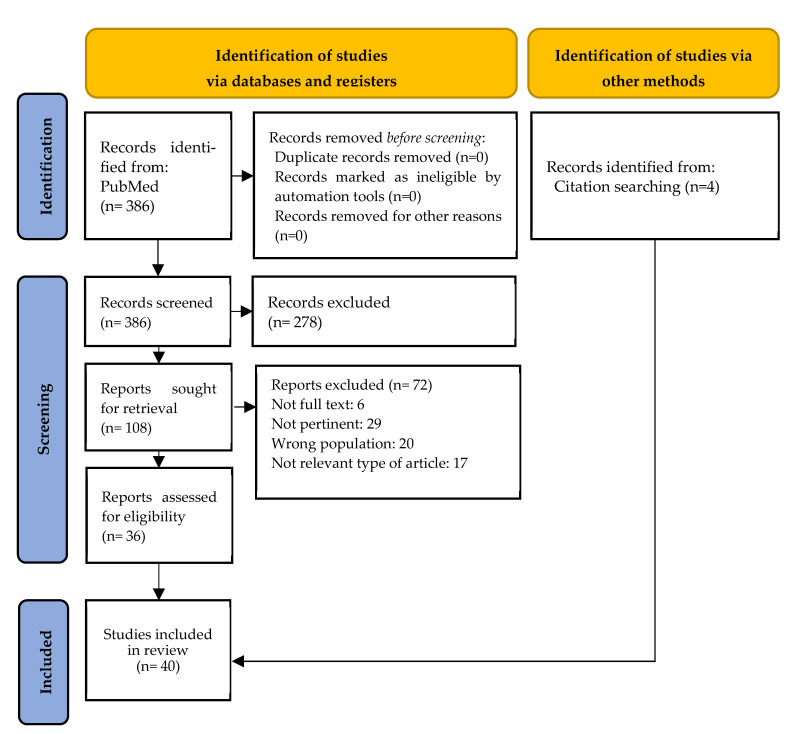
Flow chart of PRISMA study [31].

A flowchart detailing the screening process is presented in Figure 1.

The 40 studies included in the review exhibited considerable variability in methodology, geographic representativeness, and outcome focus. Approximately 43% (17/40) were based on nationally representative datasets [39,40,42,45,48,49,50,51,52,53,54,55,56,57,58,59,62], while the remaining 57% (23/40) were derived from regional networks, hospital-based registries, or single-center studies [11,14,18,22,34,35,36,37,38,41,43,44,46,47,60,61,63,64,65,66,67,68,69].

Most studies (36/40, 90%) employed an observational design, relying on direct RSV testing, typically via reverse transcription polymerase chain reaction (RT-PCR), among symptomatic adults [11,18,22,34,35,36,37,38,39,40,41,42,43,44,45,46,47,48,50,51,52,53,54,55,57,58,59,60,61,63,64,65,66,67,68,69]. These studies were predominantly conducted in hospital settings (72%, 26/36) [22,34,35,36,37,38,41,42,43,44,46,47,48,50,51,53,54,55,56,58,60,61,63,66,67,69], while a smaller proportion (28%, 10/36) assessed RSV infection prevalence and outcomes in the community or primary care settings [11,18,39,40,52,57,59,64,65,68].

A smaller subset of studies (4/40, 10%) applied modelling, meta-analysis, or time-series approaches to estimate disease burden at the population level [14,49,56,62].

The main findings from the selected studies have been described in the following two sections. The first explores the epidemiological burden of RSV, while the second examines its clinical impact, including complications, hospitalizations, and mortality among high-risk adults and elderly populations across Europe.

#### 3.1.1. The Epidemiology of RSV in the European High-Risk Adult and Elderly Population

The epidemiological burden of RSV among high-risk and older adults’ populations across Europe has been well-documented through 25 studies conducted in various settings and during different winter seasons over the past decade [11,14,18,34,35,36,37,38,39,40,41,42,43,44,45,46,47,48,62,63,64,65,66,67,68] (Tables S1 and S2).

Geographically, the majority of studies focused on Italy (8/25, 32%) [18,62,63,64,65,66,67,68,69], followed by Spain (5/25, 20%) [34,37,38,47,48], multiple European countries (4/25, 16%) [11,14,41,43], Portugal (2/25, 8%) [39,40], and Germany (2/25, 8%) [45,46]. One study each was conducted in France (1/25, 4%) [35], Belgium (1/26, 4%) [42], the Czech Republic (1/26, 4%) [44], and Greece (1/26, 4%) [36].

Approximately 28% of the studies (7/25) also provided insights into the epidemiological trends of RSV subtypes A and B circulation [18,40,44,46,47,64,65].

Significant regional variations in RSV prevalence were observed across Italy, with reported rates ranging from 4.6% to 20% [18,63,64,65,66,67,68,69]. Among individuals aged 65–80 years, prevalence varied between 8.4–9.9%, while for those over 80, it increased to 10.2–13.8% [18,63,64,65,66,67,68,69]. The distribution of RSV-A and RSV-B subtypes also showed considerable differences [18,63,64,65,66,67,68,69].

Pierangeli et al. [64] reported an overall prevalence of 18.7% for RSV-A and 23.9% for RSV-B, with an increasing trend in individuals over 80 compared to those aged 65–80 (10.2% vs. 8.4% for RSV-A; 13.8% vs. 10.1% for RSV-B).

In another study, Bracaloni et al. [18] reported that, among ARI cases in the over-65 population, the RSV-B subtype accounted for 94% of positive cases, while only 6% were attributed to RSV-A.

In Spain, there was also considerable variation in RSV prevalence among older adults, ranging from 1% [38] to 59.8% [49]. For instance, Gimferrer et al. [34], in 2015, found a prevalence of 3% among individuals over 64 years during the 2013/2014 season. A subsequent study by the same authors, in 2019 [38], examined 6534 patients aged 64 and older over five consecutive winters (2013–2017), reporting an overall prevalence of 17%, with seasonal fluctuations ranging from 1% to 29%. Higher prevalence values were observed in a study by Kestler et al. [37], which found RSV responsible for 56.8% of ARIs in individuals aged ≥65 during the 2015/2016 winter season. Similarly, Vega-Piris et al. [48] reported an RSV prevalence of 12.7% among hospitalized patients aged ≥65 during the 2021–2024 period, with a significant upward trend in RSV cases over successive seasons, peaking at 59.8% in 2023/2024.

Several other European countries have also reported notable RSV prevalence among older adults. In France, Loubet et al. [35] identified a 4.1% prevalence in hospitalized adults aged ≥65. In Portugal, Sáez-López et al. [39,40] reported a 4.7% prevalence in patients aged ≥65 over a 9-year period. Regarding the seasonal distribution of RSV subtypes A and B in patients aged ≥65 during the 2014/2015 and 2017/2018 seasons, the authors [39,40] differentiated the data based on both sentinel and non-sentinel surveillance systems. In the sentinel system, RSV-A was predominant, accounting for 64.3% (9/14) of cases. In contrast, in the non-sentinel system, RSV-B was more frequently detected, representing 71.9%, with RSV-A accounting for only 28.1% (16/57) of cases.

In Germany, Dahne et al. [45] found an RSV prevalence of 2.7% among individuals aged ≥60 with community-acquired pneumonia. In contrast, Hönemann et al. [46] reported a much higher prevalence of 64.7% among individuals aged ≥60 years between 2017 and 2023, with significant seasonal variability, including peaks of 65.6% and 78.6% in the 2017/2018 and 2018/2019 winter seasons, respectively. Overall, RSV-B was the predominant subtype (66.8%), although shifts in subtype circulation were noted during seasons overlapping with the SARS-CoV-2 pandemic. Notably, RSV-A became more prevalent in the 2019/2020 and 2021/2022 seasons (82.1% and 53.8%, respectively), while RSV-B resurged in 2022/2023, accounting for 91.1% of positive samples.

In Greece, Antalis et al. [36] estimated an RSV prevalence of 10.1% among individuals aged ≥65 across two different winter seasons (2009/2011 and 2013/2015) based on a sample of 129 patients.

High prevalence rates were also reported in Belgium, where Subissi et al. [42] found a prevalence of 32.8% among individuals aged ≥65 during the 2018/2019 winter season.

An even older population was studied by Boattini et al. [41] in Italy, Cyprus, and Portugal, where they observed a 25.9% prevalence of RSV among patients aged ≥85 years.

The only primary study (1/24, 4%) specifically focused on a high-risk adult population for RSV infection was conducted by Almeida et al. [43] and published in 2021. This study, which covered the winter seasons of 2017/2018 and 2018/2019 in Portugal, Italy, and Cyprus, included a sample of 984 adult patients (≥18 years) hospitalized or admitted to ICUs for LRTIs, reporting an RSV positivity rate of 21% (*n* = 207).

Incidence data from other countries, investigated in four out of 25 studies (4/25, 16%), further underscored the impact of RSV on older adults [11,44,47,62]. In the Czech Republic, Beran et al. [44] reported an annual incidence of 45.82 per 1000 person-years for ARIs and 30.4 for LRTIs among nursing home residents aged ≥65, with a predominance of RSV-A (71.8%). Studies conducted in Belgium, the UK, and the Netherlands [11] found RSV incidence rates ranging from 1.6–7.2%. More recently, Rojo-Alba et al. [47] reported a 1.7% incidence in individuals aged ≥70, noting a decline during the COVID-19 pandemic followed by a resurgence in 2022/2023. The results showed a consistent predominance of RSV-B, with 91 cases in the 2017/2018 season and 171 cases in 2018/2019. In contrast, RSV-A, initially less prevalent, showed a gradual increase during the SARS-CoV-2 pandemic, rising from a single case in 2018/2019 to 58 cases in 2019/2020, and 79 cases in the most recent season (2022/2023).

An Italian systematic review by Domnich et al. [62] corroborates these findings, reporting an overall RSV incidence of 1.9% in the general population aged ≥60. In high-risk groups, such as patients with cystic fibrosis or hematological conditions, incidence rates have been reported to reach as high as 10.9%.

#### 3.1.2. The Clinical Impact of RSV in the European High-Risk Adult and Elderly Population

As evidenced by 26 eligible studies analyzing complications, hospitalization rates, and mortality, RSV infections impose a significant burden on elderly and at-risk populations across Europe, placing substantial pressure on healthcare systems [11,18,22,35,41,42,44,48,49,50,51,52,53,54,55,56,57,58,59,60,61,62,64,66,69] (Tables S2 and S3).

In terms of clinical outcomes, the majority of studies (62%, 25/40) focused on hospital-based endpoints such as admission and in-hospital mortality [11,14,18,22,35,41,42,44,48,49,50,51,52,53,54,55,56,57,58,59,60,61,62,66,69], while only a few (8%, 3/40) reported on general practitioner (GP) consultations or ED attendances [11,18,57].

Regarding the geographical distribution, seven studies (7/26, 27%) reported data from multiple European countries [11,14,22,41,49,56,57], while five (5/26, 19%) focused on individuals aged over 60 in Spain [48,50,53,59,61], and another five (5/26, 19%) in Italy [18,62,64,66,69]. Additionally, four studies (4/26, 15%) examined RSV in France [35,51], two (2/26, 8%) in Denmark [52,60], and one study each investigated Belgium (1/26, 4%) [42], the Czech Republic (1/26, 4%) [44], and Germany (1/26, 4%) [55].

The majority of studies (24/26, 92%) concentrated on the elderly population aged ≥60, with the exception of one that examined at-risk adult population diagnosed with COPD [57]. Among those assessing the clinical burden of RSV in Europe, 62% (16/26) provided relevant information on the most common comorbidities among RSV-infected patients, as well as data on major complications and disease severity [11,18,22,35,44,48,51,53,54,55,56,57,58,59,60,66].

The clinical severity of RSV infection in older adults is often compounded by underlying comorbidities, particularly hypertension (46–67%), heart failure (19–48.8%), chronic obstructive pulmonary disease (COPD) (19.2–46%), diabetes (21–43%), and immunosuppression (29–40%), which contribute to a higher likelihood of complications [11,35,51,53,54]. In Italy, studies consistently highlight high rates of cardiovascular diseases (up to 69%) and chronic respiratory diseases (27–30%) in RSV-positive patients. In the study by Bracaloni et al. [18], 81.8% of 33 patients had pre-existing chronic conditions, supporting the link between comorbidities and disease severity.

Among the common complications associated with RSV infection, pneumonia is the most frequent, occurring in 24–58% of patients. Other complications include respiratory failure (29–42%), acute respiratory distress syndrome (ARDS) (13–20%), and heart failure (19–34%) [35,44,51,54]. The need for invasive or non-invasive mechanical ventilation ranges from 1.7% to 27.7% of patients, with older adults at higher risk. Loubet et al. [35] reported that 58% of hospitalized patients developed at least one complication, with pneumonia (42%), respiratory failure (29%), heart failure (19%), and ARDS (13%) being the most prevalent. Similarly, Celante et al. [51] found that 24.7% of patients were admitted to intensive care, with a significant proportion requiring mechanical ventilation or oxygen therapy. Notably, comorbidities like hypertension, chronic heart failure, COPD, and diabetes were highly prevalent in this cohort.

In Spain, Martinón-Torres et al. [53] highlighted that over 90% of patients aged 75–84 years had comorbidities, and 81.4% presented complications associated with RSV, including respiratory failure and pneumonia. These findings were echoed by Heppe-Montero et al. [50], which showed that comorbidities like hypertension and cardiovascular diseases were common among hospitalized RSV patients, contributing to the severity of the disease. Similarly, in the North Denmark Region [60], among 111 RSV positive patients with a median age of 76 years, 65% had at least one comorbidity, most commonly airway disease or cancer.

More than 60% (17/26, 65%) of these studies have reported significant hospitalization rates among older adults with RSV, showing considerable variation based on age and the presence of comorbidities [18,22,35,41,42,44,49,50,52,53,54,55,56,58,61,66,69].

The hospitalization rate for ARIs due to RSV in adults aged 65 and older shows significant variation between countries. In Spain, the hospitalization rate was 12.6%, with higher values observed in those aged ≥85 years (55.9 per 100,000 population) [50]. Additionally, another 10-year Spanish study [61] analyzing 40,600 hospitalizations due to respiratory infections in individuals aged ≥60 years revealed significant seasonal and age-related variations in RSV hospitalization incidence. Rates ranged from 21 to 406 per 100,000 person-years, with the highest incidence observed in those aged ≥80 years. The risk of RSV-related hospitalization increased with age and fluctuated considerably across different seasons, highlighting an underestimation of RSV hospitalizations by 13–40% when using the influenza-like illness (ILI) case definition compared to the combined use of ILI and extended severe acute respiratory infection (ILI/SARI) case definition.

Similarly, in Denmark and Scotland, hospitalization rates were higher in the ≥85 age group, with a rate of 7.9 per 1000 in Denmark and 8.2% in Scotland [56].

The length of hospital stays for RSV-related ARIs ranges from 6 to 18 days, depending on age group and disease severity [22,35,42,53,55]. In Germany, Mokrani et al. [54] reported an average hospital stay of 11 days for patients hospitalized with acute respiratory failure. In Italy, hospitalizations for RSV-related LRTIs were also significant, with 33.3% of the sample reporting at least one annual hospitalization, and an average length of stay of 13 days [18,62,66,69]. In France, the study by Recto et al. [58] analyzed hospital data collected over seven winter seasons (2016–2022) of 125 patients diagnosed with RSV, reporting a hospitalization rate of 83.2%, with an average length of stay of 9 days.

Mortality rates linked to RSV infection in adult and elderly patients across Europe, indagated by the 77% (20/26) also show considerable variability, ranging from 2.15% to 13%, depending on factors such as country and disease severity, and up to 36% in patients admitted to intensive care units [11,14,22,35,41,42,44,48,50,51,53,54,55,57,58,59,60,62,66,69]. In France, Loubet et al. [35] reported an 8% mortality rate in individuals aged 65 and older. Similar values were reported by Recto et al. [58], who, analyzing a population of 125 French patients aged ≥75 years with RSV positivity over the seven seasons (2016–2022) studied, estimated a mortality rate of 9.6%, while Mokrani et al. [54] documented a 13% rate among patients with a mean age of 77 years. In Northern Denmark, a study by Hagen et al. [60] on 111 RSV-positive patients with a mean age of 76 years, reported an in-hospital mortality rate of 12% for the April–December 2021 season, with no significant differences between immunocompromised patients (11%) and non-immunocompromised patients (12%).

Aligning with other European findings, in Italy, the mortality rate ranged between 7–9% in patients aged ≥65 years, with an estimate of 7.1% for individuals aged ≥70 years [69].

In Spain, a 2023 analysis revealed an overall mortality rate of 6.3%, with significantly higher rates among patients aged ≥85 (12%) and those admitted to intensive care (23%) [48,51]. Similarly, Subissi et al. [42] reported a mortality rate of 13.6% in Belgian patients aged ≥65 between 2018 and 2019. Comparable findings emerged from the Czech Republic, where the rate was 7.7% [44]. A Spanish study by Heppe-Montero et al. highlighted a sharp increase in mortality with age, reaching 36.3% in individuals over 80 and even higher rates in those with underlying conditions [50].

Lastly, a review by Savic et al. [14] emphasized that RSV-related mortality among older adults across Europe ranges from 5% to 12%, underscoring the severe impact of RSV infections on this vulnerable population.

### 3.2. An Overview of RSV Surveillance Systems: Current Practices and Approaches

Following the literature review, a structured web-based screening was conducted to collect information on international and national RSV surveillance systems, revealing a varied landscape of approaches aimed at tracking infections among high-risk and elderly populations. While this review focuses primarily on the European context, an initial overview of international RSV surveillance initiatives provides important background to understand how European systems align with global standards and to highlight where significant gaps still exist.

#### 3.2.1. The Surveillance of RSV on the International Landscape

Since 2015, the WHO has launched a global initiative to standardize RSV surveillance, building on the framework of the Global Influenza Surveillance and Response System (GISRS) [70,71]. A pilot study, initiated in 2017 across 14 countries spanning six WHO regions (including Argentina, Australia, Brazil, Canada, Chile, Côte d’Ivoire, Egypt, India, Mongolia, Mozambique, the Russian Federation, Thailand, South Africa, and the United Kingdom) [72], aimed to assess the feasibility of integrating RSV surveillance into GISRS alongside influenza. The primary goals were to enhance the understanding of RSV’s epidemiological and virological characteristics, analyze seasonality, identify high-risk groups, and measure the disease burden.

The project also focused on testing new case definitions for RSV surveillance and developing global laboratory, epidemiological, and reporting standards [70]. Many countries within GISRS started testing for RSV and other respiratory viruses as a byproduct of influenza surveillance, using the WHO-recommended case definitions for Influenza-like Illness (ILI), ARI, and Severe Acute Respiratory Infection (SARI). However, the reliance on case definitions primarily designed for other diseases, coupled with the lack of standardized testing protocols for RSV, often resulted in biased outcomes [73].

To address this, the WHO also modified the data-reporting platform for influenza (FluMart), allowing countries to upload anonymized, case-based RSV data using existing systems. Additionally, interactive data-visualization tools were developed to track trends and distributions, stratified by country, year, age, and surveillance setting [72].

The pilot program was followed by a 3-year extension phase from 2018 to 2021, expanding to over 20 countries [21]. This extension aimed to consolidate achievements, enhance surveillance capabilities, and refine RSV monitoring and data-collection systems.

In parallel, several countries have developed their own national surveillance systems for RSV and other respiratory viruses.

In the United States, the National Respiratory and Enteric Virus Surveillance System (NREVSS), coordinated by the National Center for Immunization and Respiratory Diseases (NCIRD), has been tracking viral circulation since the 1980s [74].

This system monitors a variety of viruses, including RSV, through a network of laboratories across the country, collecting data on test results, antigen detections, and polymerase chain reaction (PCR) findings. It is complemented by two additional networks: the Respiratory Virus Hospitalization Surveillance Network (RESP-NET), which tracks RSV-related hospitalizations, and the Respiratory Virus Laboratory Emergency Department Network Surveillance (RESP-LENS), which collects data from emergency departments [74].

Since 2008, the Canadian RSV Surveillance Network (CRSN) has been monitoring RSV circulation by collecting data from hospitals, clinics, and laboratories in order to monitor RSV circulation, identify regions in need of public health intervention, and guide preventive strategies and awareness campaigns [75]. Actually, the Respiratory Virus Detection Surveillance System (RVDSS) collects data from select laboratories for up to eight respiratory viruses, including RSV [75].

Meanwhile, in Australia, RSV surveillance began in 2021 under the National Notifiable Disease Surveillance System (NNDSS), which collects notifications from a range of sources, including doctors, laboratories, and hospitals. A key component of this surveillance is the Australian Sentinel Practices Research Network (ASPREN), which reports RSV and ILI seen in general practice [76,77,78].

These national surveillance systems, alongside global initiatives led by the WHO, are crucial in understanding RSV dynamics and ensuring timely, targeted public health responses. However, greater efforts are needed to establish standardized procedures worldwide to ensure the consistency and reliability of data collection, case definitions, and reporting protocols across different countries and healthcare settings.

#### 3.2.2. The Surveillance of RSV in the European Landscape

For many years, RSV surveillance in Europe has relied on multiple, often fragmented platforms, primarily focused on the pediatric population. While these systems have provided valuable insights into trends and seasonality at the national level, they have lacked comparability across countries and the ability to investigate the burden among older populations [70,79].

From 1996 to 2008, RSV data were collected and shared through the European Influenza Surveillance Scheme [70,79], a disease surveillance network primarily funded by the European Commission, based on agreed-upon surveillance recommendations [76,77,78]. The main objective of this network was to estimate the incidence of ILI during the early part of the influenza season [80,81,82].

In September 2008, following its transition to the European Centre for Disease Prevention and Control (ECDC), the network was renamed the European Influenza Surveillance Network (EISN). In collaboration with the WHO Regional Office for Europe, data collection on national RSV laboratory test results continued, but without updates to the existing surveillance recommendations [83]. In addition to laboratory data collection, nearly all EU/European Economic Area (EEA) countries have established primary care-based sentinel surveillance system (e.g., GPs and community-based pediatricians), which provides data on consultation rates for ILI and/or ARI, as well as respiratory sampling from patients across all age groups [79].

Despite these commitments, there are currently no general recommendations on RSV surveillance available in the EU for Member States that intend to establish or improve RSV surveillance.

However, in October 2023, WHO/Europe and the ECDC launched the weekly European Respiratory Virus Surveillance Summary (ERVISS), an integrated respiratory surveillance system. ERVISS provides a weekly integrated epidemiological and virological summary for influenza, RSV, and SARS-CoV-2 across the EU/EEA and the WHO European Region [83].

Despite no longer being part of the EU, the United Kingdom represents an example of a comprehensive and well-integrated RSV surveillance system. Coordinated by the UK Health Security Agency (UKHSA), it integrates and combines syndromic, laboratory, sentinel, and hospital-based data across all its four nations (England, Scotland, Wales, and Northern Ireland) [84,85]. While surveillance has traditionally focused on pediatric populations, recent efforts have expanded attention to older adults, with a notable increase in RSV hospitalization rates reported among those aged ≥65 [86,87]. This expansion aligns with the rollout of the national RSV immunization programme for adults aged 75–79 years, introduced in autumn 2024 [88].

Moreover, many other countries in Europe have established national electronic healthcare databases and registries [89], which serve as valuable tools for informing immunization policies, enhancing disease surveillance, supporting research, guiding public health decisions, planning and managing resources, and evaluating and improving healthcare services [81,89,90].

#### 3.2.3. An In-Depth Analysis of RSV Surveillance in Eight European Union Member States

An in-depth analysis was conducted through structured web screening of eight countries participating in the above-mentioned WFPHA project (Bulgaria, Finland, Germany, Italy, Norway, Portugal, Spain, and Serbia).

Overall, the findings revealed that only three of the eight (3/8, 37%) countries have implemented a structured RSV surveillance system (Table 1).

Germany, in particular, has established one of the most comprehensive RSV surveillance frameworks in Europe, combining data from sentinel physicians, hospitals, and laboratories [91]. As of 2023, laboratories are required to notify local health authorities of RSV detections under the Protection against Infection Act. Centralized at the Robert Koch Institute (RKI), both epidemiological and virological data are analyzed and disseminated through weekly reports on ARI, including RSV [91].

Similarly, Italy has made significant progress in strengthening its respiratory infection surveillance system. Beginning in the 2020–2021 season, the country transitioned from the InfluNet system, established in 1999 to monitor ILI, to the more advanced RespiVirNet system [92]. This updated framework integrates epidemiological and virological surveillance to monitor RSV, influenza, and other respiratory viruses, utilizing a network that includes GPs, Primary Care Pediatricians, Local Health Authorities, and Regional Reference Laboratories [92].

Spain also demonstrates an advanced surveillance approach, particularly in Catalonia Region, where systems like the Daily Information on Acute Respiratory Illness Plan of Catalonia (PIDIRAC) have been in place since 2006. This was further enhanced in 2022 with the launch of the Information System for the Surveillance of Infections in Catalonia (SIVIC). These systems integrate syndromic and microbiological surveillance, enabling comprehensive monitoring of RSV, influenza, and SARS-CoV-2 [93].

Across these systems, most countries continue to rely on sentinel surveillance models involving GPs and pediatricians, with Germany standing out as the only example of enhanced surveillance incorporating mandatory laboratory reporting [91,92]. Moreover, a strong pediatric focus persists, while RSV testing in older adults remains sporadic and is largely confined to hospital settings, thereby limiting the capacity to assess the community-level burden in this age group [91,92].

Despite recent advancements, routine testing of older adults for RSV across Europe remains inconsistent and predominantly restricted to severe cases in hospital settings. In most countries, RSV testing is not yet integrated into standard diagnostic protocols in primary care or outpatient settings. This fragmented approach reduces the capacity to accurately estimate the incidence and severity of RSV among older populations and likely contributes to a systematic underestimation of disease burden in this high-risk group [91,92].

Nevertheless, both Italy and Germany have recently initiated efforts to broaden RSV surveillance to better capture data in older adults in order to proactively prepare for the integration of RSV vaccination into national immunization plans. These developments reflect a growing strategic commitment to strengthening and stabilizing surveillance infrastructures in line with evolving public health priorities [91,92].

The evidence gathered for Portugal, on the other hand, indicates that it maintains a targeted RSV surveillance system focused exclusively on children under 2 years of age, with no equivalent mechanisms currently established for adult populations [94].

In contrast, the remaining countries, such as Norway, Serbia, Bulgaria, and Finland, show either limited or inconsistently implemented surveillance efforts [95,96,97,98]. This disparity underscores the urgent need to address these gaps, implementing harmonized and comprehensive approach to surveillance across Europe.

## 4. Discussion

The findings of this review underscore the substantial and growing epidemiological and clinical burden of RSV among high-risk adults and the elderly over the past decade. Consistent with previous studies [14,62], our analysis highlights the remarkable variability in RSV prevalence across Europe, with rates ranging from 1% to 64.7% [34,38] and incidence rates reaching up to 45.82 per 1000 person-years for ARIs and 30.40 per 1000 person-years for RSV-related LRTIs [44]. This variability reflects not only geographic and clinical heterogeneity but also differences in healthcare infrastructure, surveillance methodologies, and population demographics, emphasizing the complexity of RSV’s impact across different subgroups.

Of particular concern is the elevated burden of RSV in older adults, particularly those aged ≥80 years, where prevalence reaches 25.9% [41]. Additionally, in the high-risk groups with pre-existing conditions the incidence of RSV-related complications is even more pronounced, with some studies reporting rates as high as 10.9% [62].

Comorbidities such as cardiovascular diseases (69–72%), hypertension (59%), COPD (27–30%), and diabetes mellitus (15%) significantly exacerbate the severity of RSV infections [18,62,63,64,66,67,68,69]. These conditions increase the risk of severe complications such as pneumonia (24–58%), respiratory failure (29–42%), acute respiratory distress syndrome (13–20%), and heart failure (19–34%) [31,44,51,54,60]. A more detailed analysis revealed that the burden of RSV infection was particularly severe in individuals aged ≥70 years, with 69.8% of patients experiencing severe disease forms and over 33.3% requiring hospitalization [18,69]. A recent systematic review by Penders et al. [99] further highlighted the significantly increased risk of RSV-related hospitalization in adults with asthma or COPD. Among individuals aged ≥60 years, hospitalization rates reached up to 370 per 100,000 for asthma and over 1000 per 100,000 for COPD, while complication rates, including ICU admission and mechanical ventilation, frequently exceeded 20% [99].

Seasonal and regional differences in the circulation of RSV subtypes A and B add further complexity to its epidemiological landscape, influenced by factors such as climate, healthcare infrastructure, and potentially other infectious disease trends, including COVID-19 [100,101,102,103]. Traditionally, RSV follows a well-defined seasonal pattern, with epidemics occurring from November to April in the Northern Hemisphere, between August and December in equatorial regions, and from April to August in the Southern Hemisphere [100,101,103].

However, COVID-19 pandemic and the widespread implementation of non-pharmaceutical interventions, such as social distancing and mask-wearing, disrupted these seasonal trends, leading to atypical RSV circulation patterns and shifts in subtype predominance [18,39,44,46,47,64,65].

Notably, RSV-A became the predominant strain during the 2019/2020 and 2021/2022 winter seasons, and post-pandemic epidemiological shifts have been observed, with RSV-related hospitalization rates in older adults nearly doubling in the U.S. and peaking earlier than in previous years [12,18,39,44,46,47,64,65,104]. In Europe, similar disruptions occurred during the initial pandemic phase, although recent data suggest that pre-pandemic seasonal patterns and disease burden levels were largely re-established by 2022 [105]. These observations illustrate how public health interventions and behavioural changes can temporarily reshape the transmission dynamics of respiratory viruses [101], underscoring the importance of maintaining robust, population-based hospital surveillance to detect and track major shifts in the burden and epidemiological patterns of seasonal respiratory infections [106].

Beyond prevalence and clinical severity, RSV-associated hospitalizations pose a significant burden on healthcare systems. Among older adults, the hospitalization rate for RSV-related ARIs ranges from 12.6% to 55.9%, with the highest values observed in individuals aged ≥85 years [18,69]. Consistent with these findings, other studies indicate that RSV accounts for 5% to 12% of respiratory tract infections in adults over 85 years, with underreporting correction factors estimated between 3- and 49-fold in this age group [49].

The incidence of RSV-related hospital admissions among older adults is consistent across European countries, with rates of approximately 100 per 100,000 person-years in adults aged 65–74 years, 200 per 100,000 person-years in those aged 75–84 years, and 500 per 100,000 person-years in individuals aged 85 years and older [49].

Recent UK-based modelling studies have produced similar estimates, highlighting a substantial burden of RSV in older adults [49,87,105,107,108,109]. Sharp et al. [107] reported annual RSV-attributable hospitalization rates of 71 per 100,000 in individuals aged 65–74 years, increasing to 251 per 100,000 in those aged ≥75 years. Similarly, Wilkinson et al. [87] estimated that RSV accounts for approximately 175,000 GP consultations and over 15,000 hospital admissions annually among adults aged ≥60 years in England. These findings underscore the significant impact of RSV on older populations and the critical importance of targeted prevention efforts [49,87,105,107,108,109].

Mortality rates among at-risk adults and elderly patients with RSV infection across Europe also show significant variability, ranging from 2.15% to 13% [41,59], with the highest rates observed among patients requiring ICU admission [22,35,41,42,44,48,50,51,53,54,55,58,59,60].

In addition to these challenges, our findings also reveal a concerning lack of harmonization in RSV surveillance systems across the EU/EEA [70].

The COVID-19 pandemic has underscored the critical importance of robust surveillance systems for respiratory viruses, which are essential for effective healthcare planning and the timely implementation of preventive measures [110]. These systems must be meticulously designed to ensure representativeness and capable of generating accurate data that reflect both the spread and severity of respiratory pathogens [111].

In addition to tracking the intensity and distribution of infections, surveillance systems must be sufficiently sensitive to detect shifts in virus circulation patterns, monitor incidence by age group and severity, and provide crucial national and regional indicators of disease burden, such as hospitalization rates, ICU admissions, mortality, and other clinical insights [111].

Currently, RSV surveillance across the EU/EEA remains highly fragmented, posing significant challenges for effective monitoring and response.

A 2017 survey of national RSV surveillance practices within the EU/EEA revealed that 27 of 30 responding countries had some form of surveillance system in place, with half employing sentinel surveillance [79]. While these systems varied from basic aggregated data collection to more advanced case-based models, they often lacked the consistency and depth required for comprehensive epidemiological and clinical monitoring.

Since then, significant progress has been made, as evidenced by the enhanced surveillance frameworks in countries like Italy, Germany, and Spain, especially following the COVID-19 pandemic [91,103,104].

Moreover, the introduction of ERVISS system, launched by WHO/Europe and ECDC in 2023, also represents a promising step toward greater standardization, but further efforts are needed to establish a cohesive, region-wide monitoring framework to standardize practices and improve surveillance across countries [111].

One of the critical limitations of current RSV surveillance is the lack of standardized case definitions, especially in systems primarily designed for influenza surveillance. This issue complicates the differentiation between RSV and other respiratory pathogens, particularly in older adults, leading to underreporting and misclassification of cases [112].

A recent systematic literature review estimated that approximately 33% of RSV infections go undetected in adults tested for respiratory viruses [113]. Moreover, the lack of routine RSV testing in adults likely contributes to a significant underestimation of the disease burden, an issue that remains inadequately quantified in the literature [12,113,114].

This challenge is further compounded by diagnostic limitations [115]. Understanding current testing practices, including the type, timing, and combination of diagnostic specimens used, is critical to evaluate the extent of underdetection caused by suboptimal protocols [115].

Several studies have demonstrated that relying solely on nasopharyngeal swabs significantly underestimates case numbers in older adults [116,117].

In contrast, the use of multiple specimen types, such as sputum, throat, and nasal swabs, or serological testing, can markedly improve detection rates, underscoring the need for more comprehensive and standardized diagnostic approaches [116,117].

This review has several limitations. Firstly, the studies included in this review exhibit a degree of heterogeneity in terms of their designs, settings, and outcomes, which may affect the comparability of the results. Furthermore, the lack of standardized case definitions across different surveillance systems in the EU/EEA limits the comparability of data among countries and potentially reduces the reliability of the results. Additionally, there is significant underreporting of RSV cases, particularly among adults, due to the absence of routine testing for RSV in clinical settings, especially in vulnerable populations such as the elderly. Secondly, the review consulted only one database; therefore, other available studies could have been missed, and a potential selection bias could not be completely ruled out.

Another challenge is the variability in surveillance methods across countries, which ranges from aggregated data collection to more advanced case-based surveillance systems, affecting the consistency and depth of the data.

Despite these limitations, this review contributes valuable insights into the current state of RSV burden and surveillance and highlights the need for improved monitoring systems, especially for vulnerable populations.

With the advent of innovative vaccine technologies for RSV [25], the need for effective surveillance systems has become even more pressing, also in order to provide useful data for Health Technology Assessment, allowing the assessment of the overall value of available vaccines [118], as well as to plan appropriate immunization strategies capable of mitigating the RSV burden.

This need is particularly evident in countries that have already launched RSV vaccination programmes targeting older adults. A notable example is the United Kingdom, where an RSV immunisation campaign began in August–September 2024 across England, Wales, Northern Ireland, and Scotland, targeting individuals turning 75 years of age, with a catch-up campaign for those aged 75–79 years [86,88].

Early data from this rollout suggest promising reductions in RSV-related outcomes, highlighting the importance of timely implementation and continuous surveillance to assess vaccine effectiveness in real-world conditions [119,120].

In this evolving context, adaptive and resilient surveillance frameworks are essential to ensure timely, accurate public health responses. At the same time, as adult vaccination efforts expand across countries, an increase in RSV testing among older adults is expected, which must be carefully interpreted to distinguish true epidemiological trends from improved case ascertainment resulting from enhanced diagnostic activity [121].

It is also important to recognize that surveillance systems are primarily designed to monitor trends and support public health decision-making, rather than to capture every individual case. Therefore, improved surveillance must be complemented by targeted, well-designed epidemiological studies that generate robust, up-to-date estimates of RSV incidence, particularly among older adults and individuals with chronic conditions.

In the face of current diagnostic and surveillance limitations, indirect modelling approaches have played an important role in estimating RSV-attributable disease burden [107,108,109]. Building on methodologies long used for influenza, studies have generated age-stratified estimates and enabled cross-country comparisons using syndromic and virological data [107,108,109]. While these models remain indispensable in the absence of comprehensive case-based data, their accuracy may be limited by the quality of input data and could underestimate the true burden in under-tested populations [113]. As diagnostic testing improves and surveillance expands, especially among older adults, reliance on modelling may diminish. However, such approaches will continue to provide valuable support for policy planning and RSV-related resource allocation [105,107,108].

Achieving all these objectives will require a coordinated, EU-wide approach that moves beyond fragmented national efforts. Such a strategy should aim to harmonize surveillance protocols, ensure systematic inclusion of older adults and high-risk groups in both sentinel and hospital-based systems, and promote the integration of clinical and epidemiological data through interoperable national platforms with timely EU-level reporting [84]. Expanding testing frameworks, particularly among underserved populations, alongside investments in diagnostic capacity, workforce training, and technical support, would further improve data quality and representativeness [83].

While some countries have already begun tailoring global vaccination recommendations to suit their specific needs, a more unified strategy remains equally essential to ensure equitable access to vaccines for high-risk populations, particularly older adults [122]. Indeed, persistent disparities in vaccine access across EU member states, driven by differences in health system capacity, funding structures, prioritization criteria, and levels of public awareness, must be addressed through coordinated procurement mechanisms, harmonized eligibility criteria, and shared policy objectives [123].

Thus, integrating vaccination strategies with enhanced surveillance systems is crucial not only for reducing the burden of RSV but also for preparing Europe to respond effectively to future respiratory health crises. This integration will safeguard vulnerable populations, optimize vaccination strategies, and ensure a timely and efficient response to evolving health challenges.

## 5. Conclusions

RSV remains a significant, yet often underrecognized, threat to older adults and high-risk individuals. The development of new preventive tools, combined with enhanced surveillance and coordinated public health initiatives, presents a valuable opportunity to reduce the global impact of this virus. However, this will require sustained investment, international collaboration, and a commitment to evidence-based policymaking.

Investing in long-term RSV monitoring programs, potentially integrated with existing influenza surveillance efforts, will provide valuable epidemiological and clinical data to guide public health responses [122].

Strengthening RSV surveillance, particularly through the expansion of RSV-specific systems targeting vulnerable populations like the elderly, as well as expanding sentinel systems in regions where they are currently lacking, will also be crucial for ensuring comprehensive monitoring across Europe [122]. Access to data supported by strong surveillance systems, along with the ability to convert this data into meaningful insights, will empower health system decision-makers to make informed, evidence-based choices and ensure all citizens have equal access to high-quality healthcare [124].

A comprehensive strategy that prioritizes awareness, surveillance, data, and equitable access to preventive interventions will be key in mitigating the burden of RSV and improving health outcomes for vulnerable populations in the coming years.

## Figures and Tables

**Table 1:** RSV surveillance systems in eight EU member states.

Country	Presence of RSV Surveillance	Target Population	Surveillance Type
Bulgaria	None reported	-	-
Finland	None reported	-	-
Germany	Yes	All ages	Sentinel + virological data
Italy	Yes	All ages	Sentinel + virological data
Norway	None reported	-	-
Portugal	Yes	Children ≤ 2 years	Hospital sentinel + virological data
Serbia	None reported	-	-
Spain	Yes	All ages	Sentinel + microbiological data

## Data Availability

The original contributions presented in this study are included in the article. Further inquiries can be directed to the corresponding author.

## Supplementary Materials

The following supporting information can be downloaded at: https://www.mdpi.com/article/10.3390/vaccines13050510/s1. Table S1: Overview of RSV epidemiological burden results from each included primary study; Table S2: Overview of RSV epidemiological and clinical burden results from each included Systematic Review; Table S3: Overview of RSV clinical burden results from each included primary study.

## References

[1] Blount R.E., Morris J.A., Savage R. (1956). Recovery of cytopathogenic agent from chimpanzees with goryza. Proc. Soc. Exp. Biol. Med..

[2] Shi T., Denouel A., Tietjen A.K., Campbell I., Moran E., Li X., Campbell H., Demont C., Nyawanda B.O., Chu H.Y. (2020). Global disease burden estimates of respiratory syncytial virus–associated acute respiratory infection in older adults in 2015: A systematic review and meta-analysis. J. Infect. Dis..

[3] Li Y., Hodgson D., Wang X., Atkins K.E., Feikin D.R., Nair H. (2021). Respiratory syncytial virus seasonality and prevention strategy planning for passive immunisation of infants in low-income and middle-income countries: A modelling study. Lancet Infect. Dis..

[4] Kim T., Choi S.H. (2024). Epidemiology and disease burden of respiratory syncytial virus infection in adults. Infect. Chemother..

[5] Kaler J., Hussain A., Patel K., Hernandez T., Ray S. (2023). Respiratory syncytial virus: A comprehensive review of transmission, pathophysiology, and manifestation. Cureus.

[6] National Institute of Allergy and Infectious Diseases Respiratory Syncytial Virus (RSV). https://www.niaid.nih.gov/diseases-conditions/respiratory-syncytial-virus-rsv.

[7] Lai A., Bergna A., Fabiano V., Ventura C.D., Fumagalli G., Mari A., Loiodice M., Zuccotti G.V., Zehender G. (2024). Epidemiology and molecular analyses of respiratory syncytial virus in the 2021–2022 season in northern Italy. Front. Microbiol..

[8] GBD 2016 Lower Respiratory Infections Collaborators (2018). Estimates of the global, regional, and national morbidity, mortality, and aetiologies of lower respiratory infections in 195 countries, 1990-2016: A systematic analysis for the Global Burden of Disease Study 2016. Lancet Infect. Dis..

[9] Centers for Disease Control and Prevention (CDC) (2024). Surveillance of RSV. https://www.cdc.gov/rsv/php/surveillance/index.html.

[10] Falsey A.R., Hennessey P.A., Formica M.A., Cox C., Walsh E.E. (2005). Respiratory syncytial virus infection in elderly and high-risk adults. N. Engl. J. Med..

[11] Korsten K., Adriaenssens N., Coenen S., Butler C., Ravanfar B., Rutter H., Allen J., Falsey A., Pirçon J.Y., Gruselle O. (2021). RESCEU investigators. Burden of respiratory syncytial virus infection in community-dwelling older adults in Europe (RESCEU): An international prospective cohort study. Eur. Respir. J..

[12] Grace M., Colosia A., Wolowacz S., Panozzo C., Ghaswalla P. (2023). Economic burden of respiratory syncytial virus infection in adults: A systematic literature review. J. Med. Econ..

[13] Osei-Yeboah R., Spreeuwenberg P., Del Riccio M., Fischer T.K., Egeskov-Cavling A.M., Bøås H., van Boven M., Wang X., Lehtonen T., Bangert M. (2023). Estimation of the Number of Respiratory Syncytial Virus-Associated Hospitalizations in Adults in the European Union. J. Infect. Dis..

[14] Savic M., Penders Y., Shi T., Branche A., Pirçon J.-Y. (2022). Respiratory syncytial virus disease burden in adults aged 60 years and older in high-income countries: A systematic literature review and meta-analysis. Influenza Other Respir. Viruses.

[15] Nguyen-Van-Tam J.S., O’Leary M., Martin E.T., Heijnen E., Callendret B., Fleischhackl R., Comeaux C., Tran T.M.P., Weber K. (2022). Burden of respiratory syncytial virus infection in older and high-risk adults: A systematic review and meta-analysis of the evidence from developed countries. Eur. Respir. Rev..

[16] Nam H.H., Ison M.G. (2019). Respiratory syncytial virus infection in adults. BMJ.

[17] Ackerson B., Tseng H.F., Sy L.S., Solano Z., Slezak J., Luo Y., Fischetti C.A., Shinde V. (2019). Severe Morbidity and Mortality Associated with Respiratory Syncytial Virus Versus Influenza Infection in Hospitalized Older Adults. Clin. Infect. Dis..

[18] Bracaloni S., Esposito E., Scarpaci M., Cosci T., Casini B., Chiovelli F., Arzilli G., Pistello M., Panatto D., Ogliastro M. (2024). RSV Disease Burden in Older Adults: An Italian Multiregion Pilot Study of Acute Respiratory Infections in Primary Care Setting, Winter Season 2022–2023. Influenza Other Respir. Viruses.

[19] Falsey A.R., Mc Elhaney J.E., Beran J., van Essen G.A., Duval X., Esen M., Galtier F., Gervais P., Hwang S.-J., Kremsner P. (2014). Respiratory syncytial virus and other respiratory viral Infections in older adults with moderate to severe influenza-like illness. J. Infect. Dis..

[20] Du Y., Yan R., Wu X., Zhang X., Chen C., Jiang D., Yang M., Cao K., Chen M., You Y. (2023). Global burden and trends of respiratory syncytial virus infection across different age groups from 1990 to 2019: A systematic analysis of the Global Burden of Disease 2019 Study. Int. J. Infect. Dis..

[21] World Health Organization Respiratory Syncytial Virus Surveillance. https://www.who.int/teams/global-influenza-programme/global-respiratory-syncytialvirussurveillance.

[22] Boattini M., Almeida A., Christaki E., Marques T.M., Tosatto V., Bianco G., Iannaccone M., Tsiolakkis G., Karagiannis C., Maikanti P. (2021). Severity of RSV infection in Southern European elderly patients during two consecutive winter seasons (2017–2018). J. Med. Virol..

[23] Redondo E., Rivero-Calle I., Mascarós E., Ocaña D., Jimeno I., Gil Á., Linares M., Onieva-García M.Á., González-Romo F., Yuste J. (2024). Respiratory Syncytial Virus Vaccination Recommendations for Adults Aged 60 Years and Older: The NeumoExperts Prevention Group Position Paper. Arch. Bronconeumol..

[24] Anastassopoulou C., Ferous S., Medić S., Siafakas N., Boufidou F., Gioula G., Tsakris A. (2024). Vaccines for the Elderly and Vaccination Programs in Europe and the United States. Vaccines.

[25] Anastassopoulou C., Medić S., Ferous S., Boufidou F., Tsakris A. (2025). Development, Current Status, and Remaining Challenges for Respiratory Syncytial Virus Vaccines. Vaccines.

[26] European Centre for Disease Prevention and Control-ECDC RSV: Recommended vaccinations. https://vaccine-schedule.ecdc.europa.eu/Scheduler/ByDisease?SelectedDiseaseId=53&SelectedCountryIdByDisease=-1.

[27] GOV.UK RSV vaccination of older adults: Information for healthcare practitioners. Updated 25 February 2025. https://www.gov.uk/government/publications/respiratory-syncytial-virus-rsv-programme-information-for-healthcare-professionals/rsv-vaccination-of-older-adults-information-for-healthcare-practioners?utm_source=chatgpt.com.

[28] Topalidou X., Kalergis A.M., Papazisis G. (2023). Respiratory Syncytial Virus Vaccines: A Review of the Candidates and the Approved Vaccines. Pathogens.

[29] Payne A.B., Watts J.A., Mitchell P.K., Dascomb K., Irving S.A., Klein N.P., Grannis S.J., Ong T.C., Ball S.W., DeSilva M.B. (2024). Respiratory syncytial virus (RSV) vaccine effectiveness against RSV-associated hospitalisations and emergency department encounters among adults aged 60 years and older in the USA, October, 2023, to March, 2024: A test-negative design analysis. Lancet.

[30] The World Federation of Public Health Associations—WFPHA. https://www.wfpha.org.

[31] Page M.J., McKenzie J.E., Bossuyt P.M., Boutron I., Hoffmann T.C., Mulrow C.D., Shamseer L., Tetzlaff J.M., Akl E.A., Brennan S.E. (2021). The PRISMA 2020 statement: An updated guideline for reporting systematic reviews. BMJ.

[32] World Health Organization (WHO). https://www.who.int.

[33] European Centre for Disease Prevention and Control (ECDC). https://www.ecdc.europa.eu/en.

[34] Gimferrer L., Campins M., Codina M.G., Martín Mdel C., Fuentes F., Esperalba J., Bruguera A., Vilca L.M., Armadans L., Pumarola T. (2015). Molecular epidemiology and molecular characterization of respiratory syncytial viruses at a tertiary care university hospital in Catalonia (Spain) during the 2013–2014 season. J. Clin. Virol..

[35] Loubet P., Lenzi N., Valette M., Foulongne V., Krivine A., Houhou N., Lagathu G., Rogez S., Alain S., Duval X. (2017). Clinical characteristics and outcome of respiratory syncytial virus infection among adults hospitalized with influenza-like illness in France. Clin. Microbiol. Infect..

[36] Antalis E., Oikonomopoulou Z., Kottaridi C., Kossyvakis A., Spathis A., Magkana M., Katsouli A., Tsagris V., Papaevangelou V., Mentis A. (2018). Mixed viral infections of the respiratory tract; an epidemiological study during consecutive winter seasons. J. Med. Virol..

[37] Kestler M., Muñoz P., Mateos M., Adrados D., Bouza E. (2018). Respiratory syncytial virus burden among adults during flu season: An underestimated pathology. J. Hosp. Infect..

[38] Gimferrer L., Vila J., Piñana M., Andrés C., Rodrigo-Pendás J.A., Peremiquel-Trillas P., Codina M.G.C., Martín M.D., Esperalba J., Fuentes F. (2019). Virological surveillance of human respiratory syncytial virus A and B at a tertiary hospital in Catalonia (Spain) during five consecutive seasons (2013–2018). Future Microbiol..

[39] Sáez-López E., Pechirra P., Costa I., Cristóvão P., Conde P., Machado A., Rodrigues A.P., Guiomar R. (2019). Performance of surveillance case definitions for respiratory syncytial virus infections through the sentinel influenza surveillance system, Portugal, 2010 to 2018. Euro Surveill.

[40] Sáez-López E., Cristóvão P., Costa I., Pechirra P., Conde P., Guiomar R., Peres M.J., Viseu R., Lopes P., Portuguese Laboratory Network for the Diagnosis of Influenza Infection (2019). Epidemiology and genetic variability of respiratory syncytial virus in Portugal, 2014–2018. J. Clin. Virol..

[41] Boattini M., Almeida A., Christaki E., Cruz L., Antão D., Moreira M.I., Bianco G., Iannaccone M., Tsiolakkis G., Khattab E. (2020). Influenza and respiratory syncytial virus infections in the oldest-old continent. Eur. J. Clin. Microbiol. Infect. Dis..

[42] Subissi L., Bossuyt N., Reynders M., Gérard M., Dauby N., Bourgeois M., Delaere B., Quoilin S., Van Gucht S., Thomas I. (2020). Capturing respiratory syncytial virus season in Belgium using the influenza severe acute respiratory infection surveillance network, season 2018/19. Euro Surveill.

[43] Almeida A., Boattini M., Christaki E., Moreira Marques T., Moreira I., Cruz L., Tosatto V., Antão D., Bianco G., Iannaccone M. (2021). Comparative virulence of seasonal viruses responsible for lower respiratory tract infections: A southern European multi-centre cohort study of hospital admissions. Infection.

[44] Beran J., Ramirez Villaescusa A., Devadiga R., Nguyen T.L., Gruselle O., Pirçon J.Y., Struyf F., Devaster J.M. (2021). Respiratory syncytial virus acute respiratory infections in ≥65-year-old adults in long-term care facilities in the Czech Republic. Cent. Eur. J. Public. Health.

[45] Dähne T., Bauer W., Essig A., Schaaf B., Barten-Neiner G., Spinner C.D., Pletz M.W., Rohde G., Rupp J., Witzenrath M. (2024). Resurgence of common respiratory viruses in patients with community-acquired pneumonia (CAP)-A prospective multicenter study. J. Clin. Virol..

[46] Hönemann M., Maier M., Frille A., Thiem S., Bergs S., Williams T.C., Mas V., Lübbert C., Pietsch C. (2024). Respiratory Syncytial Virus in Adult Patients at a Tertiary Care Hospital in Germany: Clinical Features and Molecular Epidemiology of the Fusion Protein in the Severe Respiratory Season of 2022/2023. Viruses.

[47] Rojo-Alba S., Martínez Z.P., González-Alba J.M., Boga J.A., Varela C.O., Álvarez M.A.A., Fonseca C.P., Clemente M.M.G., Rodriguez J.G., García E.G. (2024). Respiratory syncytial virus incidence and typing in the last six seasons in the north of Spain (Asturias). Genetic characterization during the SARS-CoV-2 pandemic. J. Med. Virol..

[48] Vega-Piris L., Carretero S.G., Mayordomo J.L., Zarzuelo M.B.R., Río V.Á., García V.G., Vázquez M.G., Rodríguez M.D.C.G., Basile L., González-Coviella N.L. (2024). SARI Sentinel Surveillance Group. Severity of respiratory syncytial virus compared with SARS-CoV-2 and influenza among hospitalised adults ≥65 years. J. Infect..

[49] Johannesen C.K., van Wijhe M., Tong S., Fernández L.V., Heikkinen T., van Boven M., Wang X., Bøås H., Li Y., Campbell H. (2022). RESCEU Investigators. Age-Specific Estimates of Respiratory Syncytial Virus-Associated Hospitalizations in 6 European Countries: A Time Series Analysis. J. Infect. Dis..

[50] Heppe-Montero M., Gil-Prieto R., Del Diego Salas J., Hernández-Barrera V., Gil-de-Miguel Á. (2022). Impact of Respiratory Syncytial Virus and Influenza Virus Infection in the Adult Population in Spain between 2012 and 2020. Int. J. Environ. Res. Public Health.

[51] Celante H., Oubaya N., Fourati S., Beaune S., Khellaf M., Casalino E., Ricard J.D., Vieillard-Baron A., Heming N., Mekontso Dessap A. (2023). Prono-RSV study group of the clinical data warehouse of Greater Paris University Hospitals. Prognosis of hospitalised adult patients with respiratory syncytial virus infection: A multicentre retrospective cohort study. Clin. Microbiol. Infect..

[52] Egeskov-Cavling A.M., Johannesen C.K., Lindegaard B., Fischer T.K., PROMISE Investigators (2024). Underreporting and Misclassification of Respiratory Syncytial Virus-Coded Hospitalization Among Adults in Denmark Between 2015–2016 and 2017–2018. J. Infect. Dis..

[53] Martinón-Torres F., Gutierrez C., Cáceres A., Weber K., Torres A. (2024). How Does the Burden of Respiratory Syncytial Virus Compare to Influenza in Spanish Adults?. Influenza Other Respir. Viruses.

[54] Mokrani D., Le Hingrat Q., Thy M., Choquet C., Joly V., Lariven S., Rioux C., Deconinck L., Loubet P., Papo T. (2024). Clinical characteristics and outcomes of respiratory syncytial virus-associated ARF in immunocompetent patients: A seven-year experience at a tertiary hospital in France. J. Infect..

[55] Niekler P., Goettler D., Liese J.G., Streng A. (2024). Hospitalizations due to respiratory syncytial virus (RSV) infections in Germany: A nationwide clinical and direct cost data analysis (2010–2019). Infection.

[56] Osei-Yeboah R., Johannesen C.K., Egeskov-Cavling A.M., Chen J., Lehtonen T., Fornes A.U., Paget J., Fischer T.K., Wang X., Nair H. (2024). Respiratory Syncytial Virus-Associated Hospitalization in Adults with Comorbidities in 2 European Countries: A Modeling Study. J. Infect. Dis..

[57] Wiseman D.J., Thwaites R.S., Ritchie A.I., Finney L., Macleod M., Kamal F., Shahbakhti H., van Smoorenburg L.H., Kerstjens H.A.M., Wildenbeest J. (2024). Respiratory Syncytial Virus-related Community Chronic Obstructive Pulmonary Disease Exacerbations and Novel Diagnostics: A Binational Prospective Cohort Study. Am. J. Respir. Crit. Care Med..

[58] Recto C.G., Fourati S., Khellaf M., Pawlotsky J.M., De Prost N., Diakonoff H., Donadio C., Pouga L., de Tymowski C., Kassasseya C. (2024). Respiratory Syncytial Virus vs. Influenza Virus Infection: Mortality and Morbidity Comparison Over 7 Epidemic Seasons in an Elderly Population. J. Infect. Dis..

[59] Gomez-Garcia R.M., Jiménez-Garcia R., López-de-Andrés A., Hernández-Barrera V., Carabantes-Alarcon D., Zamorano-León J.J., Cuadrado-Corrales N., Jiménez-Sierra A., De-Miguel-Diez J. (2024). Burden of Respiratory Syncytial Virus Infection in Children and Older Patients Hospitalized with Asthma: A Seven-Year Longitudinal Population-Based Study in Spain. Viruses.

[60] Hagen T.L., Nitschke M.J., Smit J. (2025). Respiratory syncytial virus: Characteristics, complications and mortality in immunocompetent versus immunocompromised hospitalized adults in Northern Denmark. Diagn. Microbiol. Infect. Dis..

[61] Urchueguía-Fornes A., Muñoz-Quiles C., Mira-Iglesias A., López-Lacort M., Mengual-Chuliá B., López-Labrador F.X., Díez-Domingo J., Orrico-Sánchez A. (2025). Ten-Year Surveillance of RSV Hospitalizations in Adults: Incidence Rates and Case Definition Implications. J. Infect. Dis..

[62] Domnich A., Calabrò G.E. (2024). Epidemiology and burden of respiratory syncytial virus in Italian adults: A systematic review and meta-analysis. PLoS ONE.

[63] Leli C., Di Matteo L., Gotta F., Vay D., Piceghello A., Cornaglia E., Cavallo V., Busso S., Carrabba L., Mazzeo R. (2021). Prevalence of respiratory viruses by Multiplex PCR: A four-and-a-half year retrospective study in an Italian general hospital. Infez. Med..

[64] Pierangeli A., Piralla A., Uceda Renteria S., Giacomel G., Lunghi G., Pagani E., Giacobazzi E., Vian E., Biscaro V., Piccirilli G. (2023). Multicenter epidemiological investigation and genetic characterization of respiratory syncytial virus and metapneumovirus infections in the pre-pandemic 2018-2019 season in northern and central Italy. Clin. Exp. Med..

[65] Panatto D., Domnich A., Lai P.L., Ogliastro M., Bruzzone B., Galli C., Stefanelli F., Pariani E., Orsi A., Icardi G. (2023). Epidemiology and molecular characteristics of respiratory syncytial virus (RSV) among italian community-dwelling adults, 2021/22 season. BMC Infect. Dis..

[66] Santus P., Radovanovic D., Gismondo M.R., Rimoldi S.G., Lombardi A., Danzo F., Gori A., Antinori S., Rizzardini G. (2023). Respiratory syncytial virus burden and risk factors for severe disease in patients presenting to the emergency department with flu-like symptoms or acute respiratory failure. Respir. Med..

[67] Mauro M.V., Greco S., Pellegrini M., Campagna T., Caprino F., Elia N., Mastroianni A., Greco F. (2024). Epidemiology and Clinical impact of single and multi-viral respiratory infections in post-pandemic era. New Microbiol..

[68] Pierangeli A., Midulla F., Piralla A., Ferrari G., Nenna R., Pitrolo A.M.G., Licari A., Marseglia G.L., Abruzzese D., Pellegrinelli L. (2024). Sequence analysis of respiratory syncytial virus cases reveals a novel subgroup-B strain circulating in north-central Italy after pandemic restrictions. J. Clin. Virol..

[69] Cocchio S., Prandi G.M., Furlan P., Venturato G., Saia M., Marcon T., Tremolada G., Baldo V. (2023). Respiratory Syncytial Virus in Veneto Region: Analysis of Hospital Discharge Records from 2007 to 2021. Int. J. Environ. Res. Public Health.

[70] Broberg E.K., Nohynek H. (2023). Respiratory syncytial virus infections—Recent developments providing promising new tools for disease prevention. Eurosurveillance.

[71] World Health Organization (2024). Progress of Respiratory Syncytial Virus Surveillance and Disease Burden Estimation Based on the Global Influenza Surveillance and Response System: Meeting report, Lima, Peru, 22–26 April 2024.

[72] Broor S., Campbell H., Hirve S., Hague S., Jackson S., Moen A., Nair H., Palekar R., Rajatonirina S., Smith P.G. (2020). Leveraging the Global Influenza Surveillance and Response System for global respiratory syncytial virus surveillance-opportunities and challenges. Influenza Other Respir. Viruses.

[73] World Health Organization Global Influenza Programme. Respiratory Syncytial Virus Surveillance. https://www.who.int/teams/global-influenza-programme/global-respiratory-syncytial-virus-surveillance.

[74] Centers for Disease Control and Prevention National Respiratory and Enteric Virus Surveillance System (NREVSS). https://www.cdc.gov/surveillance/nrevss/index.html.

[75] Government of Canada Respiratory virus detections in Canada. https://www.canada.ca/en/public-health/services/surveillance/respiratory-virus-detections-canada.html.

[76] Sullivan S.G., Raupach J., Franklin L.J., Pennington K., Bareja C., de Kluyver R., the National Influenza Surveillance Committee, for the Communicable Diseases Network Australia (2016). A brief overview of influenza surveillance systems in Australia, 2015. Commun. Dis. Intell. Q. Rep..

[77] El Guerche-Séblain C., Rigoine De Fougerolles T., Sampson K., Jennings L., Van Buynder P., Shu Y., Sekawi Z., Yee-Sin L., Walls T., Vitoux O. (2021). Comparison of influenza surveillance systems in Australia, China, Malaysia and expert recommendations for influenza control. BMC Public Health.

[78] Immunisation Coalition Respiratory Syncytial Virus (RSV) Statistics. https://www.immunisationcoalition.org.au/news-data/respiratory-syncytial-virus-rsv-statistics/.

[79] Mollers M., Barnadas C., Broberg E.K., Penttinen P., Teirlinck A.C., Fischer T.K., European Influenza Surveillance Network (2019). Members of the European Influenza Surveillance network (EISN). Current practices for respiratory syncytial virus surveillance across the EU/EEA Member States, 2017. Euro Surveill.

[80] Meerhoff T.J., Mosnier A., Schellevis F., Paget W.J., EISS RSV Task Group (2009). Progress in the surveillance of respiratory syncytial virus (RSV) in Europe: 2001–2008. Euro Surveill.

[81] Teirlinck A.C., Broberg E.K., Stuwitz B.A., Campbell H., Reeves R.M., Carnahan A., Lina B., Pakarna G., Bøås H., Nohynek H. (2021). Recommendations for respiratory syncytial virus surveillance at the national level. Eur. Respir. J..

[82] Meerhoff T.J., Fleming D., Smith A., Mosnier A., van Gageldonk-Lafeber A.B., Paget W.J., EISS RSV Task Group (2006). Surveillance recommendations based on an exploratory analysis of respiratory syncytial virus reports derived from the European Influenza Surveillance System. BMC Infect. Dis..

[83] European Centre for Disease Prevention and Control, An agency of the European Union (2022). Operational Considerations for Respiratory Virus Surveillance in Europe. https://www.ecdc.europa.eu/en/publications-data/operational-considerations-respiratory-virus-surveillance-europe.

[84] UK Health Security Agency (2024). How We Monitor Flu and Other Respiratory Viruses Each Winter. https://ukhsa.blog.gov.uk/2024/10/08/how-we-monitor-flu-and-other-respiratory-viruses-each-winter/.

[85] GOV.UK Surveillance of Influenza and Other Seasonal Respiratory Viruses in the UK, Winter 2023 to 2024. https://www.gov.uk/government/statistics/surveillance-of-influenza-and-other-seasonal-respiratory-viruses-in-the-uk-winter-2023-to-2024/surveillance-of-influenza-and-other-seasonal-respiratory-viruses-in-the-uk-winter-2023-to-2024.

[86] Vyse A., Wright H., Begier E. (2024). Estimating adult accident and emergency attendances in English hospitals attributed to respiratory syncytial virus. Vaccine.

[87] Wilkinson T., Beaver S., Macartney M., McArthur E., Yadav V., Lied-Lied A. (2023). Burden of respiratory syncytial virus in adults in the United Kingdom: A systematic literature review and gap analysis. Influenza Other Respir. Viruses.

[88] GOV.UK (2025). Your Guide to the RSV Vaccine for Older Adults. https://www.gov.uk/government/publications/respiratory-syncytial-virus-rsv-vaccination-for-older-adults/your-guide-to-the-rsv-vaccine-for-older-adults#:~:text=From%201%20September%202024%2C%20those,cough.

[89] Pacurariu A., Plueschke K., McGettigan P., Morales D.R., Slattery J., Vogl D., Goedecke T., Kurz X., Cave A. (2018). Electronic healthcare databases in Europe: Descriptive analysis of characteristics and potential for use in medicines regulation. BMJ Open.

[90] Safran C., Bloomrosen M., Hammond W.E., Labkoff S., Markel-Fox S., Tang P.C., Detmer D.E., Expert Panel (2007). Toward a national framework for the secondary use of health data: An American Medical Informatics Association White Paper. J. Am. Med. Inform. Assoc..

[91] Robert Kock Institute How is the Activity of Acute Respiratory Infections Monitored in Germany?. https://www.rki.de/EN/Content/infections/epidemiology/inf_dis_Germany/ARI/ARI_monitoring.html?nn=7523316.

[92] Istituto Superiore di Sanità RespiVirNet. https://www.epicentro.iss.it/influenza/respivirnet.

[93] (2022). Health Information System for Surveillance of Infections in Catalonia. Gencat. https://sivic.salut.gencat.cat/documentacio.

[94] Vigilância do vírus sincicial respiratório. https://repositorio.insa.pt/bitstream/10400.18/8219/1/VigiRSV_28102021_APR.pdf.

[95] CRIStin Current Research Information System in Norway. https://app.cristin.no/projects/show.jsf?id=571616.

[96] European Commission Strengthening the Capacity of Serbia’s Health Sector for Communicable Disease Surveillance. https://www.bmeia.gv.at/fileadmin/user_upload/Zentrale/Europa/EU-Twinning/April-Juni_21/Strengthening_the_capacity_of_the_Serbia_s_health_sector_for_communicable_disease_surveillance.pdf.

[97] National Center of Infectious and Parasitic Diseases, Bulgaria. https://www.ncipd.org/index.php?option=com_content&view=featured&Itemid=730&lang=en.

[98] Finnish Institute for Health and Welfare Infectious Diseases and Vaccinations. Respiratory Virus Wastewater Monitoring. https://thl.fi/en/topics/infectious-diseases-and-vaccinations/surveillance-and-registers/wastewater-monitoring/coronavirus-wastewater-monitoring.

[99] Penders Y., Brusselle G., Falsey A.R., Rohde G., Betancur E., Guardado M.E., Agudelo J.L.R., Saeedi P., Harrington L., Michaud J.P. (2025). Burden of Respiratory Syncytial Virus Disease in Adults with Asthma and Chronic Obstructive Pulmonary Disease: A Systematic Literature Review. Curr. Allergy Asthma Rep..

[100] Kuitunen I., Renko M. (2021). Lessons to learn from the current pandemic for future non-pharmaceutical interventions against the respiratory syncytial virus—Nationwide register-study in Finland. Infect Dis..

[101] Stein R.T., Zar H.J. (2023). RSV through the COVID-19 pandemic: Burden, shifting epidemiology, and implications for the future. Pediatr. Pulmonol..

[102] Janet S., Broad J., Snape M.D. (2018). Respiratory syncytial virus seasonality and its implications on prevention strategies. Hum. Vaccines Immunother..

[103] Li Y., Wang X., Broberg E.K., Campbell H., Nair H. (2022). European RSV Surveillance Network. Seasonality of respiratory syncytial virus and its association with meteorological factors in 13 European countries, week 40 2010 to week 39 2019. Euro Surveill.

[104] Centers for Disease Control and Prevention (2022). RSV-NET Interactive Dashboard. https://www.cdc.gov/rsv/research/rsv-net/dashboard.html.

[105] Johannesen C.K., Gideonse D., Osei-Yeboah R., Lehtonen T., Jollivet O., Cohen R.A., Urchueguía-Fornes A., Herrero-Silvestre M., López-Lacort M., Kramer R. (2025). Estimation of respiratory syncytial virus-associated hospital admissions in five European countries: A modelling study. Lancet Reg. Health Eur..

[106] Havers F.P., Whitaker M., Melgar M., Pham H., Chai S.J., Austin E., Meek J., Openo K.P., Ryan P.A., Brown C. (2024). Burden of Respiratory Syncytial Virus-Associated Hospitalizations in US Adults, October 2016 to September 2023. JAMA Netw. Open.

[107] Sharp A., Minaji M., Panagiotopoulos N., Reeves R., Charlett A., Pebody R. (2022). Estimating the burden of adult hospital admissions due to RSV and other respiratory pathogens in England. Influenza Other Respir. Viruses.

[108] Fleming D.M., Taylor R.J., Lustig R.L., Schuck-Paim C., Haguinet F., Webb D.J., Logie J., Matias G., Taylor S. (2015). Modelling estimates of the burden of Respiratory Syncytial virus infection in adults and the elderly in the United Kingdom. BMC Infect. Dis..

[109] Morbey R.A., Todkill D., Watson C., Elliot A.J. (2023). Machine learning forecasts for seasonal epidemic peaks: Lessons learnt from an atypical respiratory syncytial virus season. PLoS ONE.

[110] Teirlinck A.C., Johannesen C.K., Broberg E.K., Penttinen P., Campbell H., Nair H., Reeves R.M., Bøås H., Brytting M., Cai W. (2023). New perspectives on respiratory syncytial virus surveillance at the national level: Lessons from the COVID-19 pandemic. Eur. Respir. J..

[111] European Centre for Disease Prevention and Control (2022). Operational Considerations for Respiratory Virus Surveillance in Europe. https://www.ecdc.europa.eu/sites/default/files/documents/Operational-considerations-respiratory-virus-surveillance-euro-2022.pdf.

[112] Razanajatovo N.H., Andrianirina Z.Z., Andriatahina T., Guillebaud J., Harimanana A., Ratsima E.H., Rakotoariniaina H., Orelle A., Ratovoson R., Irinantenaina J. (2021). Assessment of surveillance predictors for suspected respiratory syncytial virus, influenza and Streptococcus pneumoniae infections in children aged <5 years in Madagascar. IJID Reg..

[113] McLaughlin J.M., Khan F., Begier E., Swerdlow D.L., Jodar L., Falsey A.R. (2022). Rates of Medically Attended RSV Among US Adults: A Systematic Review and Meta-analysis. Open Forum. Infect. Dis..

[114] Widmer K., Zhu Y., Williams J.V., Griffin M.R., Edwards K.M., Talbot H.K. (2012). Rates of hospitalizations for respiratory syncytial virus, human metapneumovirus, and influenza virus in older adults. J. Infect. Dis..

[115] Kim L., Rha B., Abramson J.S., Anderson L.J., Byington C.L., Chen G.L., DeVincenzo J., Edwards K.M., Englund J.A., Falsey A.R. (2017). Identifying Gaps in Respiratory Syncytial Virus Disease Epidemiology in the United States Prior to the Introduction of Vaccines. Clin. Infect. Dis..

[116] Ramirez J., Carrico R., Wilde A., Junkins A., Furmanek S., Chandler T., Schulz P., Hubler R., Peyrani P., Liu Q. (2023). Diagnosis of Respiratory Syncytial Virus in Adults Substantially Increases When Adding Sputum, Saliva, and Serology Testing to Nasopharyngeal Swab RT-PCR. Infect. Dis. Ther..

[117] Onwuchekwa C., Moreo L.M., Menon S., Machado B., Curcio D., Kalina W., Atwell J.E., Gessner B.D., Siapka M., Agarwal N. (2023). Underascertainment of Respiratory Syncytial Virus Infection in Adults Due to Diagnostic Testing Limitations: A Systematic Literature Review and Meta-analysis. J. Infect. Dis..

[118] Calabro G.E., Carini E., Tognetto A., Giacchetta I., Bonanno E., Mariani M., Ricciardi W., de Waure C. (2022). The Value(s) of Vaccination: Building the Scientific Evidence According to a Value-Based Healthcare Approach. Front. Public Health.

[119] Hameed S.S., Robertson C., Morrison K., McQueenie R., McMenamin J., Ghebrehewet S., Marsh K. (2025). Early evidence of RSV vaccination impact on hospitalisation rates of older people in Scotland. Lancet Infect. Dis..

[120] Mensah A.A., Whitaker H., Andrews N.J., Watson C.H. (2025). Early impact of RSV vaccination in older adults in England. Lancet.

[121] Bont L., Krone M., Harrington L., Nair H., Nolan T., Oshitani H., Salisbury D. (2024). Respiratory syncytial virus: Time for surveillance across all ages, with a focus on adults. J. Glob. Health.

[122] Foundation for Infectious Disease (2022). Call to Action. Reducing the Burden of RSV Across the Life Span. https://www.nfid.org/wp-content/uploads/2023/04/NFID-RSV-Call-to-Action.pdf.

[123] Xie W., Shi L., Liu M., Yang J., Ma M., Sun G. (2024). Disparities and effectiveness of COVID-19 vaccine policies in three representative European countries. Int. J. Equity Health.

[124] de Waure C., Calabrò G.E., Ricciardi W., Value(s) of Vaccination Project Steering Committee (2022). Recommendations to drive a value-based decision-making on vaccination. Expert. Rev. Vaccines.

