# Supplementary material for: Addressing the Underestimated Burden of RSV in Older Adults in Europe: Epidemiology, Surveillance Gaps, and Public Health Implications
Source: Vaccines (Basel). 2025 May 12;13(5):510. doi: 10.3390/vaccines13050510 (PMC12115707; doi:10.3390/vaccines13050510)
Supplement: Supplementary file 1 [file vaccines-13-00510-s001.zip › vaccines-3580505-supplementary.pdf]

Supplementary Materials

Table S1. Overview of RSV epidemiological burden results from each included primary study.

FIRST AUTHOR, YEAR, COUNTRY	STUDY PERIOD AND DATA SOURCE	TARGET POPULATION	SAMPLE CHARACTERISTICS	RSV EPIDEMIOLOGICAL BURDEN		
				% RSV-A and RSV-B Subtypes	Prevalence	Incidence
**GIMFERRER L. ET AL., [34]**<br><br>**2015**<br><br>**SPAIN**	October 2013 – May 2014<br><br>Hospital data	Elderly ≥64 y	Tot: 2271 pts<br><br>Tot pts ≥64 y: 8	n.a.	8 (3%)	n.a.
**LOUBET P. ET AL., [35]**<br><br>**2017**<br><br>**FRANCE**	Season 2012–2013<br>Season 2013–2014<br>Season 2014–2015<br><br>Hospital data	Elderly pts ≥65 y with ILI	Tot hospitalized pts with ILI ≥65 y: 861 (59%)<br>Mean age: 74 y (61–84 y)	n.a.	Pts positive for ILI-RSV ≥65 y: 35/53 (66%)	n.a.
**ANTALIS E. ET AL., [36]**	Season 2009-2011<br>Season 2013-2015	Elderly ≥65 y	Tot adults ≥65 y: 129 (21.4%)	n.a.	RSV-positive adults ≥65 y: 13/129 (10.1%)	n.a.

**2018**<br><br>**GREECE**	Hospital data					
**KESTLER M. ET AL., [37]**<br><br>**2018**<br><br>**SPAIN**	Season 2015-16<br><br>Hospital data	Elderly ≥65 y with ILI	Tot ILI pts: 1,200<br>Mean age: 57.7 y	n.a.	RSV-positive ILI pts ≥65 y: 54/95 (56.8%)	n.a.
**GIMFERRER L. ET AL., [38]**<br><br>**2019**<br><br>**SPAIN**	Season 2013-2014<br>Season 2014-2015<br>Season 2015-2016<br>Season 2016-2017<br>Season 2017-2018<br><br>Hospital data	Elderly >64 y	Tot pts ≥64 years: 6.534<br><br>**Season 2013-2014**<br>Tot pts ≥64 years: 346<br><br>**Season 2014-2015**<br>Tot pts ≥64 y: 1.065<br><br>**Season 2015-2016**<br>Tot pts ≥64 y: 1.371<br><br>**Season 2016-2017**<br>Tot pts ≥64 y: 1.733<br><br>**Season 2017-2018**	n.a.	RSV-positive pts >64 y: 420 (17%)<br><br>**Season 2013-2014**<br>RSV-positive pts >64 y: 6 (1%)<br><br>**Season 2014-2015**<br>RSV-positive pts >64 y: 17 (5%)<br><br>**Season 2015-2016**<br>RSV-positive pts >64 y: 59 (12%)<br><br>**Season 2016-2017**<br>RSV-positive pts >64 y: 144 (22%)	n.a.

			Tot pts ≥64 y:<br>2.019		**Season 2017-2018**<br>RSV-positive pts<br>>64 y: 94 (29%)	
**SÁEZ-LÓPEZ E. ED AL., [39]**<br><br>**2019**<br><br>**PORTUGAL**	Season 2010-2018<br><br>National surveillance system	Elderly ≥65 y	Tot: 6,426 pts<br>Tot pts ≥ 65 y: 14.9%	n.a.	RSV-positive pts<br>≥65 y: 45/951 (4.7%)	n.a.
**SÁEZ-LÓPEZ E. ED AL., [40]**<br><br>**2019**<br><br>**PORTUGAL**	Season 2014-2015<br>Season 2017-2018<br><br>National surveillance system	Elderly ≥65 y	Tot pts ≥ 65y: 5,099	**Sentinel influenza surveillance system**<br><br>RSV-A positive pts ≥65 y: 9/14 (64.3%)<br><br>RSV-B positive pts ≥65 y: 5/14 (35.7%)<br><br>**Non-sentinel influenza surveillance system**	RSV-positive pts<br>≥65 y: 379/5,099 (7.6%)<br><br>**Sentinel influenza surveillance system**<br><br>RSV-positive pts<br>≥65 y:14 (24.6%)<br><br>**Non-sentinel influenza surveillance system**<br><br>RSV-positive pts<br>≥65 y: 57 (11.7%)	n.a.

				RSV-A positive pts ≥65 y: 16/57 (28.1%)<br><br>RSV-B positive pts ≥65 y: 41/57 (71.9%)		
**BOATTINI M. ET AL., [41]**<br><br>**2020**<br><br>**CYPRUS, ITALY, PORTUGAL**	October 2017- April 2018<br><br>October 2018- April 2019<br><br>Hospital data	Elderly ≥85 y	Tot pts ≥85 y: 251<br><br>Mean age: 89.4 ± 3.9 y (85–103)<br>M: 79 (31.5%)	n.a.	RSV-positive pts ≥85 y: 65 (25.9%)	n.a.
**SUBISSI L. ET AL., [42]**<br><br>**2020**<br><br>**BELGIUM**	Season 2018-2019<br><br>Hospital data	Elderly ≥65 y	Tot: 128 respiratory samples of pts ≥65 y	n.a.	RSV-positive pts ≥65 y: 42/128 (32.8%)<br><br>RSV-positive pts with coinfections ≥65 y: 2/128 (1.6%)	n.a.
**ALMEIDA A. ET AL., [43]**<br><br>**2021**	October 2017- April 2018<br>October 2018-April 2019<br><br>Hospital data	Hospitalized LRTI adults ≥18 y	Tot: 984 pts<br><br>Mean age: 74 y	n.a.	RSV-positive pts: 207 (21.0%)	n.a.

**CYPRUS, ITALY, PORTUGAL**						
**BERAN J ET AL., [44]**<br><br>**2021**<br><br>**CZECH REPUBLIC**	August 2003 – May 2004<br><br>August 2004 - 20 May 2005<br><br>Hospital data	Elderly ≥65 y in LTCFs	**2003-2004**<br><br>Tot pts ≥65y: 1.249<br><br>Tot pts ≥75 y: 950<br><br>**2004-2005**<br><br>Tot pts ≥65y: 1.277<br><br>Tot pts ≥75y: 993	RSV-A: 28/39 (71.8%)<br><br>RSV-B: 11/39 (28.2%)		**2004-2005**<br><br>**RSV-positive ARI cases:** 45.82 per 1,000 person-y (95% CI: 32.59–62.64)<br><br>**RSV-positive LRTI cases:** 30.40 per 1,000 person-y (95% CI: 19.86–44.54)
**KORSTEN K. ET AL.,**<br><br>**2021 [11]**<br><br>**BELGIUM, UNITED KINGDOM, NETHERLANDS**	Season 2017–2018<br>Season 2018–2019<br><br>Hospital data	Elderly ≥60 y	Tot pts ≥60 y: 1.040<br><br>F: 554 (54%)<br><br>Mean age: 75 y (60-100)<br><br>Tot pts >75 y: 562 (54%)<br><br>**Season 2017-2018**	n.a.	n.a.	**2017–2018**<br>RSV-positive pts ≥60 y: 4.2%<br><br>**2018–2019**<br>RSV-positive pts ≥60 y: 7.2%

			Tot pts ≥60 y: 527<br><br>**Season 2018-2019**<br><br>Tot pts ≥60 y: 513			Annual incidence: 1.6-7%
**LELI ET AL., [63]**<br><br>**2021**<br><br>**ITALY**	January 2016- July 2020	Elderly ≥65	Tot: 572 respiratory samples<br><br>Pts ≥60 y: 175/572 (30.6%)<br><br>Mean age: 73 y (70-79 y)	n.a.	RSV positivity in pts over 60 y: 7.4% (13/175)	n.a.
**PIERANGELI ET AL., [64]**<br><br>**2023**<br><br>**ITALY**	December 2018- 30 April 2019	Elderly ≥65 y	Tot: 11.577 respiratory samples	**RSV-A**<br>Pts ≥65 y: 51/273 (18.7%)<br><br>Aged 65–80 y: 23/273 (8.4%)<br><br>Aged ≥80 y: 28/273 (10.2%)<br><br>**RSV-B**	Pts ≥65 y: 275/1286 (21.4%)<br><br>Aged 65–80 y: 127 (9.9%)<br><br>Aged ≥80 y: 148 (11.5%)	n.a.

				Pts ≥65 y: 166/694 (23.9%)<br><br>Aged 65–80 y: 70/694 (10.1%)<br><br>Aged ≥80 y: 96/694 (13.8%)		
**PANATTO ET AL., [65]**<br><br>**2023**<br><br>**ITALY**	December 2021- March 2022	At-risk population: community-dwelling adults with respiratory symptoms	Tot: 1.213 respiratory samples<br><br>Pts aged 18–64y: 53.1% (644)<br><br>Mean age: 60.5 ± 21.5 y<br><br>F: 58.3% (707)<br><br>Pts >65 y: 46.9% (569)	**RSV-A**: 44.4%; 8/18<br><br>**RSV-B** 55.6%; 10/18	Overall RSV prevalence: 1.6%<br><br>RSV prevalence in population ≥65 y: 8/569 (1,4%)	n.a.
**SANTUS ET AL., [66]**<br><br>**2023**<br><br>**ITALY**	October 2022- March 2023	Elderly ≥65 years	Tot: 717 pts<br>M: 52.8%	n.a.	Tot RSV-positive pts: 61<br><br>Tot RSV-positive pts ≥70 y: 43 (70,5%)<br><br>Tot RSV-positive pts residing in LTCFs: 1 (1.6%)	n.a.

**DÄHNE. T. ET AL., [45]**<br><br>**2024**<br><br>**GERMANY**	July 2020 - May 2023<br><br>Hospital data	Elderly >60 y with CAP	Tot: 1.388 pts<br><br>Mean age: 68 y (67-70 y)<br><br>F: 38.1%	n.a.	RSV-positive pts ≥60 y:<br>15 (2.7%)	n.a.
**HÖNEMANN M. ET AL., [46]**<br><br>**2024**<br><br>**GERMANY**	Season 2017-2018<br>Season 2018-2019<br>Season 2019-2020<br>Season 2021-2022<br>Season 2022-2023<br><br>Hospital data	Elderly ≥60 y with RTI	Tot: 17.251 respiratory samples:	Tot<br>RSV-A: 32.9% (113/343)<br><br>RSV-B: 66.8% (229/343)<br><br>Mix: 0.3% (1/343)<br><br>**Season 2017-2018:**<br>RSV-A: 39.3% (24/61)<br><br>RSV-B: 60.7% (37/61)<br><br>**Season 2018-2019:**<br>RSV-A: 44.3% (31/70)	Tot RSV-positive samples: 477 from 343 pts<br><br>RSV-positive adults ≥60 y:<br>64.7% (222/343)<br><br>**Season 2017-2018:**<br><br>RSV-positive adults ≥60 y:<br>65.6% (40/61)<br>M: 67.2% (41/61)<br>F: 32.8% (20/61)<br><br>**Season 2018-2019:**<br><br>RSV-positive adults ≥60 y:<br>78.6% (55/70)<br>M: 48.6% (34/70)	n.a.

				RSV-B: 55.7% (39/70)<br><br>**Season 2019-2020:**<br>RSV-A: 82.1% (32/39)<br><br>RSV-B: 17.9% (7/39)<br><br>**Season 2021-2022:**<br>RSV-A: 53.8% (14/26)<br><br>RSV-B: 46.2% (26/12)<br><br>**Season 2022-2023:**<br>RSV-A: 8.2% (12/146)<br><br>RSV-B: 91.1% (133/146)<br><br>Mix: 0.7% (1/146)	F: 51.4% (36/70)<br><br>**Season 2019-2020:**<br>RSV-positive adults ≥60 y:<br>64.1% (25/39)<br>M: 56.4% (22/39)<br>F: 43.6% (17/39)<br><br>**Season 2021-2022:**<br>RSV-positive adults ≥60 y:<br>26.9% (7/26)<br>M: 57.7% (15/26)<br>F: 42.3% (26/11)<br><br>**Season 2022-2023:**<br>RSV-positive adults ≥60 y:<br>65.1% (95/146)<br>M: 52.1% (76/146)<br>F: 47.9% (70/146)
	Season 2017-2018	Elderly ≥70 y		≥70 y:	

**ROJO-ALBA S. ET AL., [47]**<br><br>**2024**<br><br>**SPAIN**	Season 2018-2019<br>Season 2019-2020<br>Season 2020-2021<br>Season 2021-2022<br>Season 2022-2023	Tot nasopharyngeal swabs collected: 191,236	Tot. 138A/401B<br><br>**Season 2017-2018**<br>-/91B<br><br>**Season 2018-2019**<br>1A/171B<br><br>**Season 2019-2020**<br>58A/26B<br><br>**Season 2020-2021**<br>-/9B<br><br>**Season 2021-2022**<br>-/20B<br><br>**Season 2022-2023**<br>79A/84B	n.a.	RSV-positive pts ≥70 y: 748/44.918 (1.7%)<br><br>**Season 2017-2018**<br>RSV-positive pts ≥70 y: 183/1998 (9.2%)<br><br>**Season 2018-2019**<br>RSV-positive pts ≥70 y:: 202/3083 (6.6%)<br><br>**Season 2019-2020**<br>RSV-positive pts ≥70 y: 150/4414 (3.4%)<br><br>**Season 2020-2021**<br>RSV-positive pts ≥70 y: 9/1497 (0.6%)

						**Season 2021-2022** RSV-positive pts ≥70 y: 21/28 372 (0.1%)<br><br>**Season 2022-2023** RSV-positive pts ≥70 y: 183/5554 (3.3%)
**VEGA-PIRIS L. ET AL., [48]**<br><br>**2024**<br><br>**SPAIN**	Season 2021-2022<br>Season 2022-2023<br>Season 2023-2024<br><br>National surveillance system	Elderly ≥65 y	Tot pts ≥65 y: 20.185	n.a.	RSV-positive pts ≥65 y: 829 (12.7%)<br><br>Season **2021-2022** RSV-positive pts ≥65 y: 66 (8%)<br><br>Season **2022-2023** RSV-positive pts ≥65 y: 267 (32.2%)<br><br>Season **2023-2024** RSV-positive pts ≥65 y: 496 (59.8%)	n.a.

**MAURO ET AL. [67]**<br><br>**2024**<br><br>**ITALY**	September 2022- March 2023	Elderly ≥65 y	Tot: 930 hospitalized pts	n.a.	Overall RSV prevalence in pts ≥65 y: 4.6%	n.a.
**PIERANGELI ET AL., [68]**<br><br>**2024**<br><br>**ITALY**	September 2021–April 2022<br><br>September 2022- April 2023.	Elderly ≥60 y	Tot: 10.689 respiratory samples	n.a.	**Season 2021-2022**<br><br>RSV-positive samples in pts ≥60 y: 34 (5.8%)<br><br>**Season 2022-2023**<br><br>RSV-positive samples in pts ≥60 y: 106 (14.8%)	n.a.
**BRACALONI ET AL., [18]**<br><br>**2024**<br><br>**ITALY**	Season 2022-2023	Elderly ≥65 y	Tot: 152 pts with ARI<br>M: 68 (44.7%)<br>F: 84 (55.3%)<br><br>Pts 65-74 y: 70 (46.0%)<br><br>Pts 75-84 y: 57 (37.5%)<br><br>Pts over 85 y: 25 (16.5%)	RSV-A: 2 cases (6.1%)<br><br>RSV-B: 31 cases (93.9%).	RSV-positive pts: 33<br><br>Pts aged 65-74 y: 13 (39.4%)<br><br>Pts aged 65-74 y: 10 (30.3%)<br><br>Pts over 85 y: 10 (30.3%)	n.a.

* ARI: Acute Respiratory Infection
CAP: Community-Acquired Pneumonia
ILI: Influenza-Like Illness
LRTI: Lower Respiratory Tract Infection
LTCFs: Long term care facilities
n.a.: Not available
Pts: Patients
Y: Years

Table S2. Overview of RSV epidemiological and clinical burden results from each included Systematic Review.

FIRST AUTHOR, YEAR, COUNTRY	NUMBER OF INCLUDED STUDIES	TARGET POPULATION	RSV EPIDEMIOLOGICAL AND CLINICAL BURDEN
Savic M. et al., [14] 2022<br><br>High-income countries (U.S., Canada, European countries, Japan, South Korea)	21	Elderly ≥60 y	**Epidemiological burden**<br><br>**Europe**<br>- RSV-ARI attack rate: 3.05% – 7.2%<br><br>**European countries: Germany, Italy, France, Spain, UK (2019 population data)**<br>- RSV-ARI attack rate: 1.62%<br><br>**Clinical burden**<br><br>**Europe**<br>- RSV-ARI hospitalization rate: 0.31% (95% CI: 0.085–0.800)<br>- RSV-ARI in-hospital mortality rate: 9.8% – 13.6%

			**European countries: Germany, Italy, France, Spain, UK (2019 population data)**<br>- RSV-ARI hospitalization rate: 0.15%<br>- RSV-ARI in-hospital mortality rate: 7.13%
Domnich et al., [62]<br>2024<br><br>Italy	37	Elderly ≥60 y<br>At-risk adults	**Epidemiological burden**<br><br>- RSV Cumulative incidence in community-dwelling adults aged ≥60 y: 1.9%<br>- RSV positivity prevalence in adults ≥60 y: 4.4%<br>- RSV attack rate in outpatients with hematologic malignancies: 10.9% (Symptomatic: 85.7%; Asymptomatic: 14.3%)<br><br>- RSV attack rate in cystic fibrosis pts: 4.4%<br>- RSV positivity prevalence in immunocompromised pts: 11.5%<br><br>**Clinical burden**<br><br>Antibiotic use among RSV-positive pts:<br>- Age group 65–80 y: 63.3%<br>- Age group ≥80 y: 79.3%

	Use of mechanical ventilation (invasive/non-invasive) among hospitalized RSV-positive pts ≥65 y: 27.3%<br><br>In-hospital mortality:<br>- Age group ≥65 y: 9.1%<br>- Age group ≥70 y: 7.1%<br><br>Mean hospital length of stay in patients ≥65 y: 23 days

* ARI: Acute Respiratory Infection

Pts: Patients

Y: Years

Table S3. Overview of RSV clinical burden results from each included primary study.

FIRST AUTHOR, YEAR, COUNTRY	STUDY PERIOD AND DATA SOURCE	TARGET POPULATION	CHARACTERISTICS OF RSV-POSITIVE SAMPLE	HEALTHCARE RESOURCE USE	HOSPITALIZATIONS	MORTALITY
**LOUBET P. ET AL., [35]**<br><br>**2017**	Season 2012-2015<br><br>Hospital data	Elderly ≥65 y	RSV-positive pts with ILI: 53<br><br>Mean age: 74y (61-84)	n.a.	Mean hospital length of stay: 9 days<br><br>ICU admissions: 8 pts (15%)	RSV-related deaths: 4/53 (8%)

**FRANCE**						
**BOATTINI M. ET AL., [41]**<br><br>**2020**<br><br>**CYPRUS, ITALY, PORTUGAL**	October 2017-April 2018<br><br>October 2018-April 2019<br><br>Hospital data	Elderly ≥85 y	RSV-positive pts ≥85 y: 65 (25.9%)	n.a.	Mean hospital stay for community-acquired Influenza A/B and/or RSV: 12 ± 11.1 days<br><br>Mean hospital stay of hospital-acquired Influenza A/B and/or RSV: 27.8 ± 28.7 days	Pts with community-acquired Influenza A/B and/or RSV: 31 (14%)<br><br>Pts with hospital-acquired Influenza A/B and/or RSV: 4 (13.3%)
**SUBISSI L. ET AL., [42]**<br><br>**2020**<br><br>**BELGIUM**	Season 2018-2019<br><br>Hospital data	Elderly ≥65 y	RSV-positive pts ≥65 y: 42 (18.1%)	n.a.	Mean hospital stay for pts ≥65 y: 9.5 days	Mortality among RSV-positive pts ≥65 y: 6 (13.6%)
**BERAN J. ET AL., [44]**<br><br>**2021**<br><br>**CZECH REPUBLIC**	Season 2003-2004<br>Season 2004-2005<br><br>Hospital data	Elderly ≥65 y	2004-2005<br><br>RSV-ARI episodes: 39 (11.64%)<br><br>RSV-LRTI episodes: 26 (11.98%)	n.a.	Hospitalizations: 4 (10.3%)<br>Median hospital stay: 18.5 days (: 14.0–37.0)	RSV-ARI-related deaths: 3 (7.7%)

**BOATTINI M. ET AL., [22]**<br><br>**2021**<br><br>**CYPRUS, ITALY, AND PORTUGAL**	Season 2017-2018<br><br>Hospital data	RSV hospitalized elderly ≥65 y	RSV-positive pts ≥65 y: 166<br><br>Mean age:<br>80.9 ± 8.7 y<br>M: 63 (38%)<br><br>Aged 65-74 y: 41<br>M: 24 (58.5%)<br><br>Aged 75-84 y: 62<br>M: 25 (40.3%)<br><br>Aged ≥85 y: 63<br>M: 14 (22.2%)	Invasive ventilation in pts ≥65 y: 16.3%	Mean length of hospital stay for pts ≥65 y: 11.8 ± 12.2 days<br><br>Aged 65–74 y: 10.9 ± 9.8 days<br><br>Aged 75–84 y: 13.9 ± 16.5 days<br><br>Aged ≥85 y: 10.4 ± 7.4 days	Deaths among RSV-positive pts ≥65 y: 20 (12.1%)<br><br>Aged 65–74 y: 6 (14.6%)<br><br>Aged 75–84 y: 5 (8.1%)<br><br>Aged ≥85 y: 9 (14.3%)
**KORSTEN K. ET AL., [11]**<br>**2021**<br><br>**BELGIUM, UNITED KINGDOM, THE NETHERLANDS**	Season 2017–2018<br><br>Season 2018–2019<br><br>Hospital data	Elderly ≥60 y	2017-2018<br><br>Tot RSV-ARTI positive pts: 22<br><br>2018-2019<br><br>Tot RSV-ARTI positive pts: 37	Any medication: 10 (28%)<br><br>Respiratory drugs: 9 (25%)<br><br>Antibiotics: 2 (6%)<br><br>Medical visit: 11/36 (31%)<br><br>GPs visit: 10 (28%)	n.a.	0 (0%)

				Phone call to physician: 2 (6%)		
**JOHANNESEN C.K. ET.AL., [49]**<br><br>**2022**<br><br>**DENMARK, UNITED KINGDOM, FINLAND, NORWAY, NETHERLANDS, SCOTLAND**	Season 2006-2018<br><br>National registry and laboratory-based data	Elderly ≥60 y	n.a.	n.a.	**Pts aged 65-74 y**<br><br>**Scotland**<br>RSV incidence rate: 0.6/1,000<br><br>**United Kingdom**<br>RSV incidence rate: 0.9/1.000<br><br>**Netherlands**<br>RSV incidence rate: 0.6/1.000<br><br>**Norway**<br>RSV incidence rate: 1.6/1.000<br><br>**Pts aged 75-84 y:**<br><br>**Scotland**<br>RSV incidence rate: 3.0/1.000<br><br>**United Kingdom**	n.a.

					RSV incidence rate: 2.8/1.000<br><br>**Netherlands**<br>RSV incidence rate: 1.3/1.000<br><br>**Pts ≥85 y:**<br><br>**Scotland**<br>RSV incidence rate: 5.0/1.000<br><br>**United Kingdom**<br>RSV incidence rate: 6.0/1.000<br><br>**Netherlands**<br>RSV incidence rate: 3.1/1.000<br><br>**Finland**<br>RSV incidence rate: 0.2/1.000<br><br>**Denmark**<br>RSV incidence rate: 0.1/1.000	
	Season 2012-2020	Elderly ≥60 y with RTI	Tot RSV-RTI hospitalizations in	n.a.		In-hospital RSV mortality in pts ≥60y (per

**HEPPE-MONTERO M. ET AL., [50]**<br>**2022**<br>**SPAIN**	Hospital data		pts ≥60 y: 17.312 (12.6%)<br>Mean age: 65 y (31-81 y)		Tot RSV-RTI hospitalizations in pts ≥60 y: 17.312 (12.6%)<br>RSV hospitalization rate (per 100,000 inhabitants)<br>Pts aged 60-69 y: 3023 (2.2%)<br>Annual Incidence rate: 0.67 (0.66-0.68)<br>Pts aged 70-79 y: 4830 (3.5%)<br>Annual Incidence rate: 1.48 (1.47-1.49)<br>Pts 80-89 y: 7065 (5.1%)<br>Annual Incidence rate: 3.44 (3.41-3.47)<br>Pts ≥90 y: 2394 (1.7%)<br>Annual Incidence rate: 5.56 (5.49-5.63)	100,000 inhabitants): 7.91%<br>Pts aged 60-69 y: 179 (10.8%)<br>Annual Incidence rate: 5.92<br>Pts aged 70-79y: 305 (18.5%)<br>Annual Incidence rate: 6.31<br>Pts aged 80-89 y: 600 (36,3%)<br>Annual Incidence rate: 8.49<br>Pts aged ≥90 y: 286 (17.3%)<br>Annual Incidence rate: 11.95
**CELANTE H. ET AL., [51]**	Season 2015-2019	Elderly ≥65 y	Tot RSV-positive hospitalized pts ≥65 y: 1,168	Oxygen therapy: 18.2%	ICU admissions: 288 (24.7%)	Overall mortality in RSV-positive pts ≥65 y: 77 (6.6%)

**2023**<br>**FRANCE**	Hospital data		Median age: 75 y (63-85 y)<br>F: 631 (54%)	Non-invasive ventilation: 15.2%<br>Invasive ventilation: 7.4%<br>Antibiotic use: 82.1%		Pts 65-74 y: 12 (5%)<br>Pts 75-84 y: 14 (6%)<br>Pts ≥85 y: 41 (12%)<br>Mortality in ICU for RSV-positive pts ≥65 y: 37 (12.8%)
**EGESKOV-CAVLING A.M. ET AL., [52]**<br>**2023**<br>**DENMARK**	Season 2015-2016<br>Season 2017-2018<br>National registries and microbiological database	Elderly ≥66 y with RSV-RTI	Tot RSV-positive pts 66–75 y: 279<br>Tot RSV-positive pts ≥75 y: 425	n.a.	RSV hospitalization rate<br>Pts 66–75 y: 119/279 (42.7%)<br>Pts ≥75 y: 179/425 (42.1%)	n.a.
**COCCHIO ET AL., [69]**<br>**2023**<br>**ITALY**	January 2007-December 2021	Elderly ≥65 y	Tot RSV-positive pts >70 y: 64 (15.9%)	n.a.	Hospitalizations: 2.4% (169)	Deaths among RSV-positive pts over 70 y: 56.5% (3/23)
**PIERANGELI ET AL., [64]**	December 2018- April 2019	Elderly ≥65 y	Tot RSV-positive respiratory samples: 275	Antibiotic therapy	n.a.	n.a.

**2023**<br><br>**ITALY**	(analisi stagionale)			Aged 65–80 y: 63.3%<br><br>Aged 80 y: 79.3%		
**SANTUS ET AL., [66]**<br><br>**2023**<br><br>**ITALY**	October 2022- March 2023<br><br>seasonal analysis	Elderly ≥65 y	RSV-positive pts: 61<br><br>M: 26 (42.6%)	**Outpatient treatments**<br><br>Bronchodilators: 20 (33.3%)<br><br>Inhaled corticosteroids: 17 (28.3%)<br>Immunosuppressants: 5 (8.3%)<br><br>Systemic steroids: 4 (6.7%)<br><br>**In-hospital treatments**<br><br>Antibiotics: 47 (79.0%)<br><br>Systemic steroids: 40 (65.6%)	≥1 hospitalizations: 19 (33.3%)<br><br>Non-severe RSV infection: 6 (35.3%)<br>Severe RSV infection: 13 (32.5%)<br><br>Mean hospital stay: 13 days	In-hospital deaths: 4 (6.6%)<br><br>Deaths in ICU: 4 (6.6%)

				Inhaled steroids: 42 (70.0%)<br><br>Oseltamivir: 8 (13.1%)<br>Other antivirals: 5 (8.2%)		
**MARTINON-TORRES F. ET AL., [53]**<br><br>**2024**<br><br>**SPAIN**	2015<br>2017<br>2018<br><br>Hospital data	Elderly ≥ 65 y	Tot pts ≥65 y: 2,085<br><br>Tot pts 65-74 y: 480<br><br>Tot pts 75-84 y: 796<br><br>Tot pts ≥ 85 y: 809	n.a.	Mean RSV hospitalization rates (per 100,000 elderly population)<br><br>Pts ≥65 y: 23.7/100.000<br><br>Pts 65–74 y: 10.8/100.000<br><br>Pts 75–84 y: 26.7/100.000<br><br>Pts ≥85 y: 55.9/100.000<br><br>ICU admission rates	Mortality rates<br><br>Pts ≥65 y: 7.2%<br><br>Pts 65–74 y: 5.0%<br><br>Pts 75–84 y: 7.5%<br><br>Pts ≥85 y: 8.2%<br><br>ICU mortality rates<br><br>Pts ≥65 y: 23%<br><br>Pts 65–74 y: 14.7%<br><br>Pts 75–84 y: 27.9%

					Pts ≥65 y: 10.4%<br>Pts 65–74 y: 11.5%<br>Pts 74-84 y: 11.1%<br>Pts >85 y: 1.3%	Pts ≥85 y: 38.1%
**MOKRANI D. ET AL., [54]**<br>**2024**<br>**FRANCE**	October 2016-March 2023<br>Laboratory and hospital data	Elderly ≥60 y	Tot hospitalized RSV-ARI pts: 104<br>M: 49 (47%)<br>Mean age:77 y (67-85 y)	Ventilatory support: 25%<br>Non-invasive mechanical ventilation: 17%<br>Invasive mechanical ventilation: 6%	Tot hospitalized RSV-ARI pts: 104<br>Mean hospital stay: 11 days<br>ICU admission: 20%<br>Mean ICU stay: 4 days	30-day mortality: 13% (14/104)
**NIEKLER P. ET AL., [55]**<br>**2024**<br>**GERMANY**	2010-2019<br>National registry data	Elderly ≥60 y	Total RSV-positive pts: 5,900<br>Median age: 79 y (r 72-85 y)	n.a.	Hospitalized RSV-positive pts: 2.9%<br>Median hospital stay: 8 days	In-hospital mortality: 458 (7.8%)

			F: 3,319 (56.3%)		ICU admissions: 640 (10.8%)	
**OSEI-YEBOAH R. ET AL., [56]**<br><br>**2024**<br><br>**DENMARK, SCOTLAND**	Denmark (2010-2018)<br><br>Scotland (2010-2016)<br><br>National hospital registries and virological surveillance data	Elderly ≥65 y with comorbidities	n.a.	n.a.	Hospitalization rates<br><br>Denmark<br>Aged 65–74 y: 2.0% (1.7-2.3)<br><br>Aged 75–84 y: 4.9% (4.2-5.6)<br><br>Aged ≥85 y: 7.9% (6.7-9.1)<br><br>Scotland<br><br>Aged 65–74 y: 0.9% (0.6-1.2)<br><br>Aged 75-85 y: 3.0% (2.0-3.6)<br><br>Aged ≥85 y: 8.2% (5.2-9.6)	n.a.
**VEGA-PIRIS L. ET AL., [48]**<br><br>**2024**<br><br>**SPAIN**	Season 2021-2022<br><br>Season 2022-2023	Elderly ≥65 y	Tot pts ≥65 y: 20,185<br>RSV-positive pts ≥65 y: 829<br><br>Mean age: 83 y (75–89)	n.a.	ICU admission: 3.8%	Mortality: 6.3%

	Season 2023-2024<br><br>National surveillance system		F: 513 (61.9%)<br><br>M: 316 (38.1%)			
**WISEMAN D.J. ET AL, [57]**<br><br>**2024**<br><br>**UNITED KINGDOM, NEATHERLAND**	Season 2017-2018<br><br>Season 2018-2019<br><br>Season 2019-2020<br><br>Laboratory and hospital data	Adults ≥40 y with COPD	Tot RSV pts: 377<br><br>United Kingdom: Tot:177<br><br>Mean age: 72.11 y<br>M: 63%<br><br>Netherlands<br>Tot: 200<br>Mean age: 67.6 y<br>M: 69%	Outpatient visits: 1,999	n.a.	Death number: 15
**RECTO C. G. ET AL., [58]**<br><br>**2024**<br><br>**FRANCE**	Season 2016-2022<br><br>Hospital data	Elderly ≥75 years	Tot RSV-positive pts: 125<br><br>Mean age: 85.5 y<br>M: 39.2%	Antibiotic therapy: 47.2%	Hospitalization rate: 83.2%<br><br>Mean length of stay: 9 days<br><br>ICU admission: 7.2%	Mortality: 9.6%

**GOMEZ-GARCIA R. M ET AL., [59]**<br><br>**2024**<br><br>**SPAIN**	2016-2022<br><br>Hospital data	Elderly ≥65 years with asthma	Tot hospitalized patients ≥65 y with RSV and asthma: 2,389	Invasive ventilation: 1.72%<br><br>Non-invasive ventilation: 4.69%	ICU admission by year<br><br>2016: 11.83%<br>2017: 7.84%<br>2018: 4.12%<br>2019: 5.26%<br>2020: 3.46%<br>2021: 5.69%<br>2022: 2.7%	Mortality by year<br><br>2016: 2.15%<br>2017: 5.88%<br>2018: 6.53%<br>2019: 5.06%<br>2020: 5.68%<br>2021: 9.76%<br>2022: 5.01%
**BRACALONI ET AL. [18]**<br><br>**2024**<br><br>**ITALY**	Season 2022-2023<br><br>Seasonal analysis	Elderly ≥65 y	RSV-positive pts: 33	Initial antibiotic treatment: 15.1%<br><br>Antibiotics (general use): 59.2%<br><br>Aerosol therapy: 2.6%<br><br>Corticosteroids and paracetamol: 22.2%<br><br>**Other healthcare resource use**	Hospitalizations after ED visit: 1 pts	n.a.

				Contact with GP: 74%<br><br>Outpatient visits: 32%<br><br>Specialist consultations: 16%<br><br>ED access: 6%<br><br>Hospitalizations after ED visit: 1 pts		
**HAGEN T.L. ET AL., [60]**<br><br>**2025**<br><br>**DENMARK**	April-Dicember 2021<br><br>Hospital data	Elderly ≥60 y	Tot hospitalized pts: 111<br><br>Mean age: 76 (63-82) y<br><br>F: 53%	Oxygen therapy: 68%<br>Non-invasive ventilation: 3% (n = 3)<br>Invasive ventilation: 3% (n = 3)<br><br>Antibiotic therapy: 86% (n = 96)	n.a.	In-hospital mortality: 12%
**URCHUEGUÍA-FORNES A. ET AL., [61]**<br><br>**2025**	Season 2010/2011-2019/2020<br><br>Hospital data	Elderly ≥60 y	Tot: 790<br>F: 448 (57%)<br><br>Mean age: 81 y	n.a.	**Incidence rates by ILI/SARI/RSV**<br><br>**General population over 60 y**	n.a.

**SPAIN**		Pts aged ≥70 y: 668 (85%)<br><br>Pts aged ≥80 y: 432 (55%)	Range: 30.3 (season 2013/14) to 154.3 (season 2016/17) per 100,000 person-years<br><br>**Population aged ≥80 y**<br>Range: 44.4 (season 2013/14) to 406.3 (season 2016/17) per 100,000 person-years<br><br>**Incidence rates by ILI-RSV definition**<br><br>**General population over 60 y**<br>Range: 21.1 (season 2013/14) to 112.9 (season 2018/19) per 100,000 person-y<br><br>**Population aged ≥80 y**<br>Range: 27.7 (season 2013/14) to 300.9 (season 2018/19) per 100,000 person-y

*ARI: Acute respiratory infections

COPD: Chronic obstructive pulmonary disease

ED: Emergency department

GP: General practitioner

ICU: Intensive care unit

ILI: Influenza-like illness

LRT: Lower respiratory tract

Pts: Patients

Y: Years
